# A New Approach to Model Pitch Perception Using Sparse Coding

**DOI:** 10.1371/journal.pcbi.1005338

**Published:** 2017-01-18

**Authors:** Oded Barzelay, Miriam Furst, Omri Barak

**Affiliations:** 1 School of Electrical Engineering, Faculty of Engineering, Tel-Aviv University, Tel Aviv, Israel; 2 Rappaport Faculty of Medicine, Network Biology Research Laboratories, Technion, Haifa, Israel; University of Tübingen and Max Planck Institute for Biologial Cybernetics, GERMANY

## Abstract

Our acoustical environment abounds with repetitive sounds, some of which are related to pitch perception. It is still unknown how the auditory system, in processing these sounds, relates a physical stimulus and its percept. Since, in mammals, all auditory stimuli are conveyed into the nervous system through the auditory nerve (AN) fibers, a model should explain the perception of pitch as a function of this particular input. However, pitch perception is invariant to certain features of the physical stimulus. For example, a missing fundamental stimulus with resolved or unresolved harmonics, or a low and high-level amplitude stimulus with the same spectral content–these all give rise to the same percept of pitch. In contrast, the AN representations for these different stimuli are not invariant to these effects. In fact, due to saturation and non-linearity of both cochlear and inner hair cells responses, these differences are enhanced by the AN fibers. Thus there is a difficulty in explaining how pitch percept arises from the activity of the AN fibers. We introduce a novel approach for extracting pitch cues from the AN population activity for a given arbitrary stimulus. The method is based on a technique known as sparse coding (SC). It is the representation of pitch cues by a few spatiotemporal atoms (templates) from among a large set of possible ones (a dictionary). The amount of activity of each atom is represented by a non-zero coefficient, analogous to an active neuron. Such a technique has been successfully applied to other modalities, particularly vision. The model is composed of a cochlear model, an SC processing unit, and a harmonic sieve. We show that the model copes with different pitch phenomena: extracting resolved and non-resolved harmonics, missing fundamental pitches, stimuli with both high and low amplitudes, iterated rippled noises, and recorded musical instruments.

## Author Summary

By means of a sound's pitch, we can easily discern between low and high musical notes, regardless of whether they originate from a guitar, piano or a vocalist. The relation between different sounds that yield the same percept is what makes pitch an interesting subject of research. Today, despite extensive research, the mechanism behind this physical to perceptual transformation is still unclear. The large dynamic range of the cochlea combined with its nonlinear nature makes the modeling and understanding of this process a challenging task. Given a large amount of physiological and psychological data, a general explanation consistent with many of these phenomena would be a major step in elucidating the nature of pitch perception. In this paper, we recast the problem in the general framework of sparse coding of sensory stimuli. This framework, initially developed for the visual modality, posits that the goal of the neural representation is to represent the flow of sensory information in a concise and parsimonious way. We show that applying this principle to the problem of pitch perception can explain many perceptual phenomena.

## Introduction

The perception of pitch is an important feature of speech recognition and perception of musical melodies. It conveys information of prosody and speaker identity; it helps in grouping different tones into one auditory object; and it conveys information about melody and harmony. The sensation of a pitch, usually associated with the periodicity of a given physical stimulus, is usually perceived as having two dimensions: pitch class and pitch height. The pitch class, or the pitch chroma, is the set of all pitches that are related by whole octave numbers and is known in musical theory as "octave equivalence"; the pitch height is the continuum perception of sound from low to high. The percept of pitch is so inherent in us that usually even a slight repetition in time is needed to create it. When dealing with harmonic signals, pitch is usually related to the first harmonic, the fundamental frequency, of that signal. Even though most natural sounds are not strictly periodic, pitch is still clearly perceived and used by the brain in various hearing-related tasks. A unique property of pitch perception is that it is a many-to-many mapping: a similar pitch can be perceived by different acoustic stimuli, and a given acoustic stimulus can yield different percepts of pitch. This property is the reason that makes pitch an interesting property of the mind, but it is also the reason that makes it hard to explain. The question arises: How does a brain manage to perform this task?

For almost a century, pitch properties have been extensively researched both experimentally and theoretically. Generally, most of the existing models that have emerged from this research activity can be divided into two main categories: (1) temporal models and (2) spectral models [1]. Modern temporal models, which are currently regarded as prominent, are usually based on autocorrelation principles [2–4]. These models rely on the fact that periodic stimuli, with the same perceived pitch but with possibly different spectral harmonic content, have the same temporal periodic response. For example, a signal consisting of the first six consecutive harmonics will have the same temporal period as a signal that contains just three successive harmonics. Thus, both signals are likely to reveal the same perception of pitch. The predictions of the temporal models are consistent with a large number of psychoacoustic properties of pitch perception, including: (a) the missing fundamental case, also known as virtual pitch, which is the pitch of a harmonic series that does not include its fundamental frequency; (b) the pitch shift effect, which is the perception of a signal with shifted, equally spaced, harmonic components that yield ambiguous pitches [5]; and (c) the invariance to the stimuli amplitude levels, which is an inherent property of the autocorrelation process, in accordance with psychophysical measurements [6]. There is also neurophysiological evidence for reliably predicting pitches for different stimuli [7,8] based on calculations of the autocorrelation of a cat’s AN population response⁠. On the other hand, it seems that temporal models perform too well compared to human psychophysics. Consider for example the case of resolved and unresolved stimuli. Low harmonics are known to be resolved, meaning they are transformed into distinct rate activity within AN fibers and with distinct peaks at certain CFs. On the other hand, higher harmonics, approximately the 5^th^ to 10^th^ and above [9], are unresolved in the sense that having these harmonics in the same stimulus they share the same spatial area along the cochlea. Temporal models cope well with both types of stimuli [10] because autocorrelation accounts for the interaction between the different harmonic components of the signal. However, previous measurements have shown that stimuli composed of resolved (low) harmonics are usually more salient than stimuli that are composed of unresolved (high) harmonics [11,12]. Another example of the excessive performance of these temporal models over human performance is the case of the transposed tones [13]. Transposed tones of low harmonic stimuli are designed to have, using modulation, the same auditory peripheral representation as their low harmonic counterparts. These experiments suggest that temporal information alone is not sufficient and that tonotopic organization must be considered [14]⁠. Finally, temporal models require certain physiological structures in the auditory neural pathway to work. In particular, the autocorrelation functionality requires the existence of (at least) 40ms long tapped delay lines [15]. But at present there is currently no physiological evidence to support such mechanism.

A second major class of models is the spectral theory for pitch. These models are based on the tonotopic organization, or mapping, from stimulus frequencies to stimulated spatial locations along the cochlea; high frequencies resonate the basal parts of the cochlea while low frequencies resonate its apical parts. These vibrations are transduced into the auditory system through the innervation of the auditory nerve (AN) fibers. The spatial arrangement of these ANs along the cochlea means that each of these neurons is most responsive to a specific frequency, which is denoted as its characteristic frequency (CF). Spectral models exploit this mapping to extract the frequency components of the incoming stimuli. A prominent implementation of these models is the class of pattern-matching models [16–18]. The overall structure shared by these types of models is composed of two main phases: the first extracts the spectral components of the stimulus from the AN population activity, and the second matches the resulting spectral pattern with the model's existing templates. Each of these templates is indexed to match different pitches, and a percept of a particular pitch is the best probable match between a given stimulus and a certain template.

Similarly to the temporal models, the predictions of these models are also consistent with a large number of psychoacoustic properties of pitch [19–22]. However, there are also psychoacoustic phenomena that are difficult to explain within this framework. One main disadvantage of these models is their inability to infer pitches that are composed of high harmonic components. As mentioned above, the cochlea decomposes the stimulus’ frequencies into spatial locations, which are represented by the CFs, and not all harmonics transduce into auditory activities in the same way. As a result, spectral models cannot easily account for pitches of unresolved stimuli.

Common to these models is the use of the AN population response to extract features from a given stimulus. These features are then translated into a scalar that represents the pitch percept. The problem of feature extraction from input stimuli has been studied in more general settings [23,24], and it is instructive to consider this approach in a wider sense. The general task of all modalities is the need to process streams of incoming sensed data abundant with information, and to extract desired low dimensional properties from it. For example, in the visual system a low dimensional percept of an object’s orientation is extracted from the activities of photoreceptors in the retina at the time of the stimulus (high dimensional input). Likewise, in the case of the auditory system, the input signal is a continuous auditory stream. It is composed of the spiking activity of approximately 30,000 ANFs in a healthy human adult and lasts for the duration of the whole stimulus. Yet, the auditory system usually extracts relatively low-dimensional and slow changing features such as the pitch of that signal. Namely, the perception of the pitch height and the pitch class is represented by just two dimensions as opposed to about 30,000 dimensions for each ANF that changes over time.

Today there are well developed and closely related mathematical frameworks that specialize in feature extraction. Specifically, we refer to a family of algorithms known as *sparse coding* (SC). This mathematical technique has many applications displaying a wide variety of variants and flavors [25]. Additionally, it seems that the SC approach is in accordance with the known physiology of the central nervous system, that of the auditory system and of other physiological modalities [23].

In this paper we apply a SC algorithm to predict pitches for different signals. We apply it to the AN population responses taken from simulations of known cochlear models [26–29]. The SC algorithm concurrently uses both the spatial and temporal domain. In this sense, the proposed model is a hybrid of the “classic” temporal and the spectral pitch models mentioned above. We show that this type of model can predict a variety of psychoacoustic properties of pitch. Specifically, the model can infer the pitches of missing fundamental complex tones; it exhibits the psychoacoustic phenomena of pitch shifts; and it is invariant to stimuli levels. Our results suggest that the principle of sparse coding can explain relatively high perception functionality such as pitch.

### Model Overview

**Fig 1:**
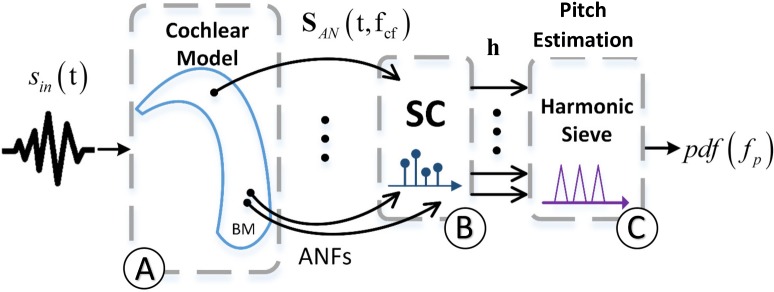
The model. The model is composed of three main sections: **(A)** The cochlear model [26–29] transduces a one-dimensional input stimulus, s_in_(t), into a two-dimensional matrix that represents the AN population response, **S**_AN_(t,f_CF_). **(B)** The AN’s spatiotemporal response is introduced into the sparse coding (SC) block to produce the sparse coefficient vector, **h**. The vector **h** carries invariant information of the input stimulus that we refer to as pitch cues. The (sparse) information in **h** represents harmonics in s_in_(t). **(C)** Finally, the likelihood probability of the pitch given the vector **h** is extracted and denoted as *pdf*(*f*_*p*_).

The proposed model consists of three main parts: (i) a cochlear model that translates auditory stimulus, s_in_(t), into the AN population activity, **S**_AN_(t,f_CF_) (Fig 1A); (ii) a sparse coding (SC) unit that represents the AN population response as a sparse (few non-zeros) set of coefficients, **h**. (Fig 1B); (iii) a readout unit that translates the active coefficients in **h** into a probability density function *pdf*(*f*_*p*_) of pitches. To compare the model with known psychoacoustic phenomena, we set the estimated stimulus’ pitch to be the maximum value of that pdf function, s_p_ ∈ *R*^1^ (Fig 1C). This scalar represents the most plausible pitch for the particular given input signal, s_in_(t). Each part is briefly described in the following sections.

#### The Cochlear Model

For low and medium sound levels, each location along the BM represents a specific frequency, the CF of this location. We denote these frequencies by f_CF_. The cochlear models that we use in this paper [26–29] transduce a stimulus, ${\text{s}}_{\text{in}}\left(\text{t}\right)\in R^{{\text{T}}_{\text{a}}}$, into instantaneous rates of the ANs, **S**_AN_(t,f_CF_) ∈ *R*^T×N^. Accordingly, T_a_ is the total length of the stimulus in samples; T is a segment out of T_a_ for the cochlear response; and N is the number of the CF channels in the cochlear model. Throughout this paper, regardless of the cochlear model that we use, we always set T = 5ms; this 5ms interval is taken from the end of the total T_a_ = 15ms cochlear simulation.

**Fig 2:**
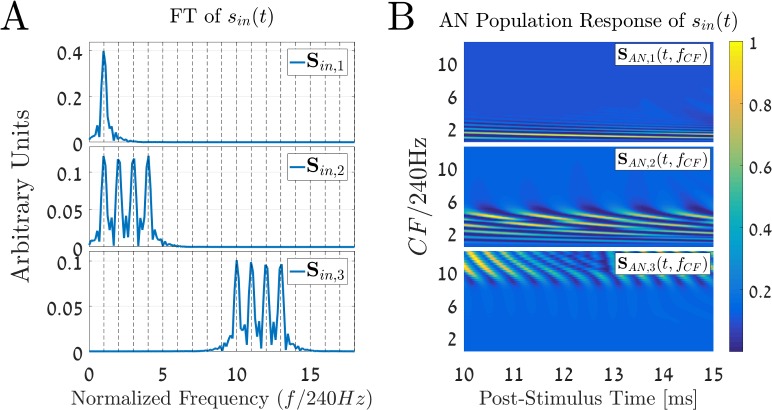
Different complex harmonic stimuli with the same pitch. (A) The Fourier transform (FT) of three complex harmonics stimuli with a fundamental frequency of f_0_ = 240 Hz. The three signals have different spectral components: **S**_in,1_(f) is composed of the first harmonic component of f_0_; **S**_in,2_(f) consists of the first four successive harmonics of f_0_; and **S**_in,3_(f) is formed from of the 10–13 harmonics. (B) The corresponding output of the cochlear model [26], i.e., the AN population responses for the three stimuli. The y-axis represents the normalized characteristic frequencies (CFs), which is CF divided by f_0_, on a linear scale, and the x-axis shows the post-stimulus time in milliseconds. The cochlear input is a 15ms long stimulus, and the resulting output is taken from the last 5ms. Note the different patterns of the AN activities that correspond to the three different cases: a stimulus with low frequencies excites the apical parts of the cochlea (lower part in the images), while a stimulus with higher frequencies excites the basal parts. Note also that the AN population responses define unique spatiotemporal patterns of activities for each of the stimuli. All the three stimuli have relatively low sound levels (30 dB SPL), which means that the cochlea response is linear.

Fig 2B shows the cochlear model [26] responses **S**_AN_(t,f_CF_) for the following three stimuli

$$\begin{array}{l} s_{in,1}\left(t\right)=g_{30dB}\cdot \sin\left({2\pi \text{kf}}_{0}\text{t}\right)\\ s_{in,2}\left(t\right)=g_{30dB}\cdot \sum_{k=1}^{4}\sin\left({2\pi \text{kf}}_{0}\text{t}\right)\\ s_{in,3}\left(t\right)=g_{30dB}\cdot \sum_{k=10}^{13}\sin\left({2\pi \text{kf}}_{0}\text{t}\right) \end{array}.$$ (1)

The parameter f_0_ = 240 Hz is the fundamental frequency of the given harmonic series. The amplitude of each of the stimuli, g_30dB_, is given in Pascals and is equivalent to 30 dB SPL in this case. Fig 2A represents the Fourier transform (FT) of these three stimuli, **S**_in,r_(f) = FT{s_in,r_(t)}, which denote the three different cases for r ∈ {1, 2, 3}, respectively. The first stimulus, **S**_in,1_(f), consists of the first fundamental component, f_0_. The second stimulus, **S**_in,2_(f), includes the first four successive harmonics of f_0_, and **S**_in,3_(f) contains the 10^th^ to 13^th^ harmonics of f_0._ Note that although these stimuli sound differently, it is known [9,30,31] that these stimuli, especially the first and second ones, yield the same percept of pitch, namely f_0_.

The normalized AN population responses, **S**_AN,r_(t,f_CF_), of all the three stimuli, r ∈ {1, 2, 3}, are depicted in Fig 2B. The matrix **S**_AN,r_(t,f_CF_) is presented in the figure as a color-coded image, where the x-axis represents the post-stimulus time (10ms to 15ms), and the y-axis represents the normalized CFs relative to the stimuli’s fundamental frequency, i.e., f_CF_ /f_0_. It is clear from Fig 2B that the AN population responses depend on the spectrum of the input signal. For instance, the **S**_AN,1_(t,f_CF_), that corresponds to the 240 Hz sine wave, shows local AN activities only in lower CFs (at the apical part of the cochlea). On the other hand, the **S**_AN,3_(t,f_CF_), which is composed of the 10–13 harmonics of f_0_, yields activity at higher CFs (towards the basal part of the cochlea).

It is noteworthy that each frequency component in the auditory stimulus reflects the ANs activity of specific location along the cochlea. Hence, each of these AN population responses has its own spatiotemporal typical pattern.

#### The Sparse Coding Phase

Next, we wish to exploit the unique aforementioned spatiotemporal structures of the AN population responses. Given the AN response to a specific stimulus, we wish to represent it as a weighted sum of a small number of response primitives using the following optimization:

$$\arg\;\underset{h}{\min}\;\frac{1}{2}{\left\|v_{\text{AN}}-Dh\right\|}_{2}^{2}+\lambda {\left\|h\right\|}_{1}^{},$$ (2)

Where the operator ∥∙∥_2_ specifies the Euclidian norm, ∥⋅∥_1_ is the ℓ_1_-norm. The vector **v**_AN_ ∈ *R*^*T*∙*N*^ is the vectorized AN response; the matrix **D** ∈ *R*^*T*∙*N*×*M*^ is a collection of M primitives known as the dictionary; the vector **h** ∈ *R*^*M*^ is the (sparse) coefficient vector; and *λ* is a scalar that controls the sparseness of the solution **h**. Note that the entries in **h** assign weights to different atoms in **D**, and thus we have *h*[*k*] ≥ 0 for all *k* ∈ [1,*M*]. There are various numerical techniques [25] to solve Eq 2 for **h**; here we chose to use LASSO, a linear regression with ℓ_1_-norm regularization [32,33].

The dictionary **D** can either be learned from examples [24,34–37], or chosen according to some prior knowledge (see for example [25], Ch. 12). In this paper, we opt for the latter option but will also explore the effect of a different dictionary later on. Thus, we chose those primitives, which within the SC paradigm are known as atoms, to be the AN population response to pure sine waves,

$$s_{d}\left(t\right)=g_{30dB}\cdot \sin\left({2\pi \text{f}}_{d}\text{t}+\phi_{g}\right).$$ (3)

In this equation, *d* ∈ [1,*gM*] is the index of the atom, each one is created by its own *s*_*d*_(*t*) stimulus; the parameter f_*d*_ ∈ [100 *Hz*, 20*k Hz*] is the frequency that this particular atom represents. When solving for Eq 2, we may need the dictionary to account for different phases of an incoming stimulus. One simple way to achieve that is to use groups *g* ≥ 1 of atoms with the same frequencies f_*d*_ but with different phases *ϕ*_*g*_ = 2*π*/*g* ⋅ *k*, *k* ∈ [0,*g*−1]. This technique can be seen as a simplified variant of the group-lasso algorithms [38,39]. Solving for Eq 2, the solution of the sparse coefficient vector $\tilde{h}\in R^{gM}$ contains entries that belong to the same group (same frequency, different phases). These entries represent the same frequency and should not “compete”. Thus, the entries of each group are summed together, forming the final sparse vector **h** ∈ *R*^*M*^. In the following, we would assume that *g* = 1 for simplicity (i.e., each atom is a group that is created by a sine with zero phase). We would use the extended scheme (*g* > 1) when dealing with stimuli of random or unknown phase (see Iterated Rippled Noise and Musical Notes).

**Fig 3:**
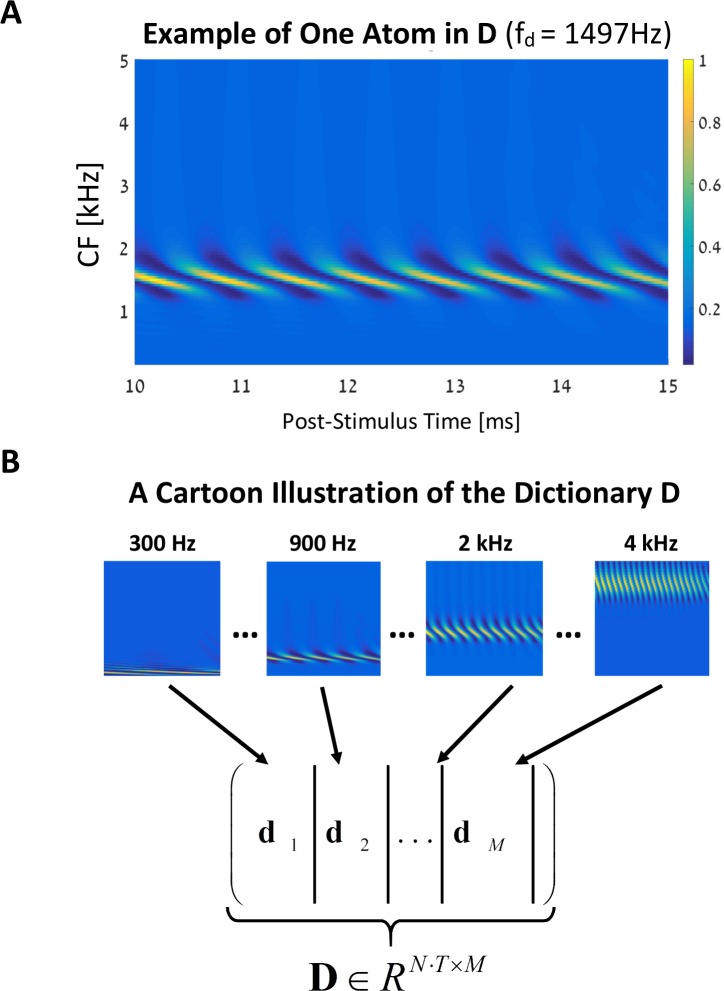
Composing the dictionary D. **(A)** An example of one atom in **D**. It is the AN population response to a sine wave of 1.5k Hz tone generated by the cochlear model [26]. The atom was normalized to a peak value of 1, as for all other population responses. The y-axis of the two-dimensional matrix represents the CF along the BM, and the x-axis is the post-stimulus time in milliseconds. **(B)** Each of the atoms, **d**_j_, j ∈ [1, M], is vectorized into a column in **D**. These M columns are concatenated to form the dictionary matrix **D**. All the input signals used for the creation of the dictionary have the same level of 30 dB SPL (i.e., at the cochlear linear region). In this example, we used only one atom per group (*g* = 1).

Fig 3A shows an example of an atom **d** ∈ *R*^*T*⋅*N*^, *g* = 1, that corresponds to a sinusoid stimulus of about *f*_*d*_ = 1.5*k Hz*. Fig 3B shows a typical dictionary that is a concatenation of M such atoms, i.e., **D** = [**d**_1_,…,**d**_*M*_]. As mentioned, the scalar *λ* = 0.01 in Eq 2 determines the sparseness of the solution **h**. On the one hand, increasing *λ* assigns more weight to the ℓ_1_-norm (the second term in Eq 2) and so leads to a sparser solution for **h** (i.e., more components in **h** are set to zero). But this sparseness comes at the expense of the matching between **v**_AN_ and **Dh** (the first term in Eq 2) and for a large enough *λ* the solution becomes trivial, **h** = 0. On the other hand, setting *λ* = 0.0 usually leads to a non-sparse solution, that is, most of the entries in **h** are nonzero. With regard to this observation, the solution of Eq 2 decreases to an ordinary least-square (LS) solution, without any sparseness considerations.

**Fig 4:**
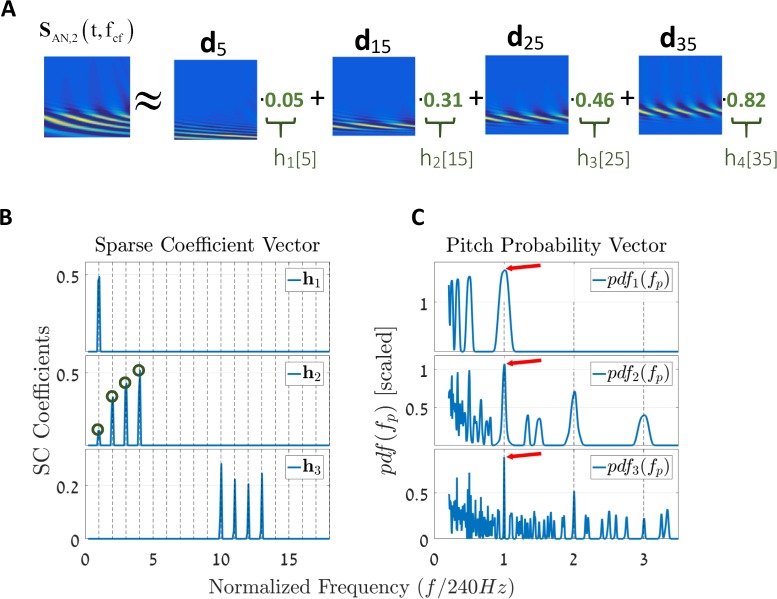
The sparse coefficient vector h and the final pitch probability vector. **(A)** A simplified view of the SC methodology. The algorithm decomposed the two-dimensional signal **S**_AN,2_(t,f_CF_) into a linear combination of four atoms (columns) within **D**. This is a simplified view that shows the primary values in **h**_2_ (green indices) multiplied by the atoms. **(B)** The sparse coefficient solution vectors, **h**_k_, for the three cases (k ∈ [1,2,3]). The green circles in the figure of **h**_2_ correspond to the four terms in the simplified example of (A). All x-axes are normalized by the fundamental frequency f_0_ = 240 Hz for convenience. Observe that the solutions for **h**_k_ resemble that of the FT for the respective stimuli (**Fig 2**A). **(C)** Using the pitch estimation unit (harmonic sieve), we can easily map the information in **h**_k_, for k ∈ [1,2,3], into a pitch probability vector, *pdf*(f_p_). Each of the y-axes of the pdfs functions is multiplied by a constant (x100) for visual clearance. The red arrows indicate the locations of the maximum peaks, all of which are shown to occur at the fundamental harmonic. In other words, it is most probable that all three stimuli represent the same pitch. Still, note that other options are also plausible, especially in rational ratios of f_0_.

As a simplified example, consider the AN population response of the second stimulus case (Fig 2B). Solving for **h**, the AN population response is equivalent to a linear combination of just four atoms in **D**, that is **v**_*AN*_ ≈ 0.05 ⋅ **d**_5_ + 0.31 ⋅ **d**_**1**5_ + 0.46 ⋅ **d**_**2**5_ + 0.82 ⋅ **d**_**3**5_ (Fig 4B, green circles in **h**_2_). Solving for Eq 2 for the other three signals, we get the solutions of **h**_1_, **h**_2_, and **h**_3_, respectively (Fig 4B). Note that there is a clear similarity between the FT of the input signals (Fig 2A) and the derived coefficients of **h**_1_, **h**_2_, and **h**_3_. Explicitly, the spectral structures of the stimuli are reconstructed by the SC algorithm, yet one can see that the three expressions are not alike. For example, there is a difference between the magnitudes of the FT coefficients of s_in,2_(t) and the nonzero entries in **h**_2_, meaning that the SC decomposition is not in general an FT decomposition.

#### Pitch Estimation

The output of the SC stage is a sparse vector **h** (Fig 4B) that represents the weights of each atom in the dictionary **D**. We wish to relate a single pitch percept for each such vector to facilitate a comparison with human psychophysics' tests. We do this by computing the likelihood for each possible pitch [16,19,40], by assuming a generative model of a harmonic series for each pitch. The resulting likelihood function is a normalized product of the vector **h** with a template for the specific pitch in question:

$$pdf\left(f_{P}\right)=\frac{1}{\left\|G\cdot \tilde{h}\right\|}G\left(f_{p},\sigma_{p}\right)\cdot \tilde{h},$$ (4)

where $\tilde{h}$ is an interpolated version of the vector **h** (see Methods); each row of the matrix **G** (*f*_*p*_,*σ*_*p*_) ∈ *R*^*P*×*P*^ contains Gaussian functions centered around the harmonics of the pitch *nf*_*p*_ as weights; and $\left\|G\cdot \tilde{h}\right\|$ is the normalization factor of the pdf. The derived probability density functions *pdf*_*r*_(*f*_*p*_), for r ∈ {1, 2, 3}, are depicted in Fig 4C. The three pdfs are aligned, and the x-axis is normalized by the fundamental frequency *f*_*o*_ for convenience. The red arrows show that the maximum peaks in each of the three cases are pointing at *f*_*o*_. Note that the maximum peak in the probability distribution is just one option among many. For example, in the first case, in which s_in,1_(t) is composed of just the fundamental spectral component, the *pdf*_1_(*f*_*p*_) indicates that other pitches are also possible; specifically, these other options occur at subharmonics of *f*_*o*_. Adding spectral components into the stimulus, i.e., in the second case of *pdf*_2_(*f*_*p*_), narrows the width of the peaks. Additionally, more peaks appear at harmonics that are not complete ratios of *f*_*o*_. In the third case of s_in,3_(t), which contains the 10^th^-13^th^ harmonics, the probability function is denser. This thickening of the pdf indicates that the third stimulus is perceived as having less salience than the other two (for a detailed treatment of salience, see Resolved and Unresolved Harmonics). Additional peaks are formed (not shown, but see the same effect in Resolved and Unresolved Harmonics) in its probability function, *pdf*_3_(*f*_*p*_), around the ‘center-of-mass’ of the spectral components (i.e., the f_locus_ around the 10^th^-13^th^ harmonics[1]).

Finally, a standard paradigm in psychoacoustic experiments is to yield a scalar, i.e., specific pitch or a pitch difference, per a given stimulus. Moreover, it is a known practice in psychoacoustics to restrict the participants (or to modulate the results) to a one octave interval (see for example [41–43]). Thus, the collapsing of the resulting probability function, the *pdf* (*f*_*p*_), into a single scalar, the inferred pitch, is a straightforward and convenient procedure that enables us to compare the model at hand with known psychophysical results. In this paper we defined the estimated pitch for a given stimulus as

$${\hat{f}}_{o}=\underset{f_{p}}{\max}\left\{pdf\left(f_{p}\right)\right\}.$$ (5)

Additionally, in some of the displayed cases, we follow the convention by limiting the inferred pitch to an octave around the fundamental frequency.

## Results

Below, we demonstrate the ability of the proposed model to match known psychoacoustic phenomena qualitatively. Using these phenomena, we illustrate how the various components of the model contribute to its performance.

### Why Do We Need Sparse Representation?

**Fig 5:**
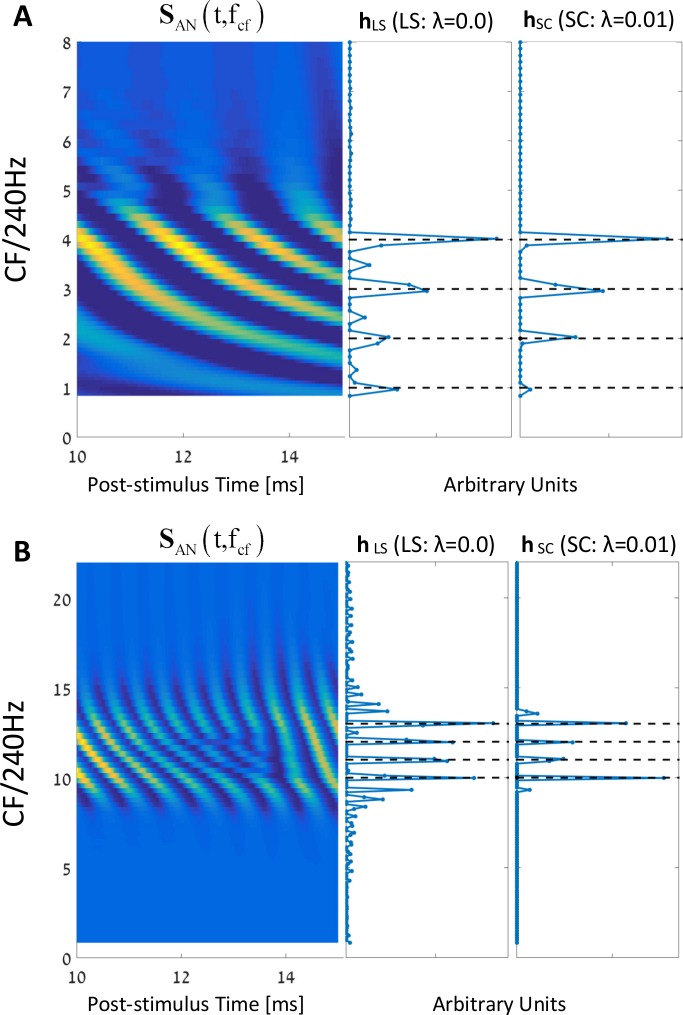
Comparing LS with SC. **(A)** From left to right: the AN population response for a harmonic complex with the 1^st^ – 4^th^ harmonics. The y-axis is the CFs normalized by the fundamental frequency, in a linear scale (f_0_ = 240 Hz). The x-axis indicates the post-stimulus time (between 10ms to 15ms). Next, the **h** coefficient vectors for the LS case (λ = 0.0) and for the sparse case (λ = 0.01). **(B)** Same as in (A) but for a complex tone stimulus that contains the harmonics 10^th^–13^th^. Note that for the lower harmonic stimulus (A), the results between the two cases, i.e., **h**_LS_ vs. **h**_SC_, are almost identical. On the other hand, for the stimuli with the higher harmonics (B), the difference is more substantial. Specifically, there are much more nonzero coefficients in **h**_LS_ than in **h**_SC_ that are unrelated to the original spectrum structure of the signal (compare with the FTs in **Fig 2**A).

To demonstrate the advantage of using sparse coding algorithms, we compared the performance of the algorithm (Eq 2) for sparse (λ = 0.01) and non-sparse solutions (λ = 0, Least squares). The resulting vectors, **h**_*LS*_ and **h**_*SC*_, for the two cases are shown in Fig 5 for the two aforementioned 30 dB SPL stimuli of *s*_*in*,2_(*t*) and *s*_*in*,3_(*t*). In the case of *s*_*in*,2_(*t*), there is little difference between the two solutions (Fig 5A). For the case of *s*_*in*,3_(*t*), which includes relatively higher (non-resolved) frequency components, the difference is more substantial (Fig 5B). Still, although the LS solutions (**h**_*LS*_) yield more nonzero coefficients than those of the sparse ones (**h**_*SC*_), the solutions can visibly be related to the harmonic structures of the two input stimuli (compare the resulting **h** vectors with the FTs in Fig 2B).

**Fig 6:**
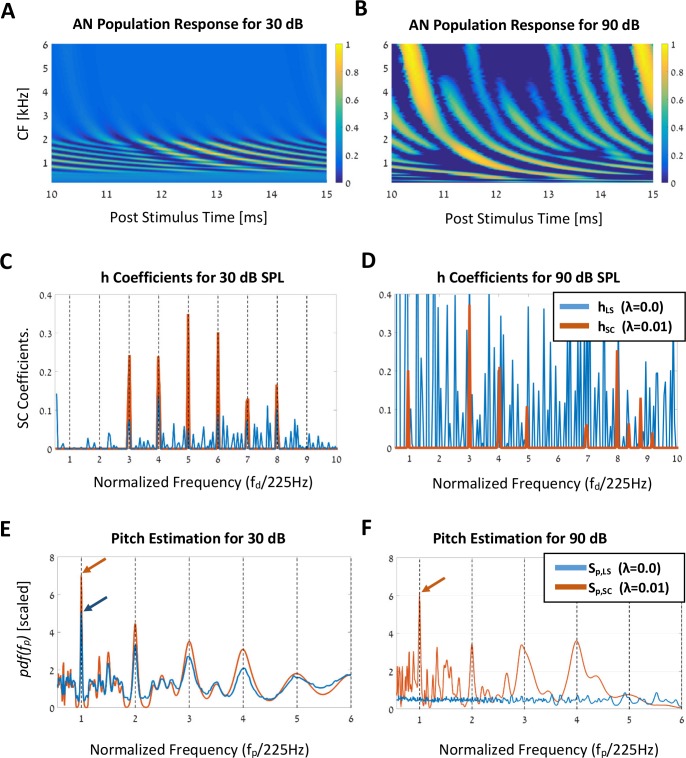
Stimulus level invariance. **(A)** The AN population response for the missing-fundamental harmonic complex tone of Eq 6, with f_0_ = 225 Hz. The stimulus has an amplitude level of 30 dB SPL, and the AN population response is normalized to one, as usual. The x-axis shows post-stimulus time, and the y-axis denotes the (linear) mapping between locations along the cochlea and CFs. **(B)** The AN population response for the same spectral structure as in **A** (3–8 harmonics), but for a stimulus level of 90 dB SPL. For this relatively high stimulus level, the nonlinearity effects of the cochlea over the AN population response are apparent. **(C–D)** The solutions of the LS case (**h**_LS_) and the SC case (**h**_SC_) for the 30 dB (C) and 90 dB SPL (D) stimulus levels, respectively. **(E–F)** Probability functions of the LS (**S**_p,LS_) and the SC (**S**_p,SC_) cases, for the two amplitude levels, respectively. In the 30 dB SPL case (E), the same pitch is succesfully estimated for both the LS and the SC simulations (blue and red arrows indicate maximum peaks). However, for the 90 dB SPL case (F) only the SC solution proved to be robust and invariant to the stimulus level, as desired (red arrow indicates maximum peaks). In order to account for the cochlear nonlinearities due to the changing in the stimuli levels, all simulations of the AN fibers in this section were made using Carney's cochlea model (Zilany’s et al. [27–29]).

The benefits of the sparse representation are evident when we introduce stimuli with high volume levels. Fig 6 compares the processing of a missing fundamental harmonic series with six harmonic components (k ∈ [3,8]),

$$s_{in,L}\left(t\right)=g_{L}^{}\cdot \left(\sum_{k\in \left[3,8\right]}\sin\left(2\pi \cdot {\text{f}}_{0}\text{k}\cdot \text{t}\right)\right).$$ (6)

The fundamental frequency is set to f_0_ = 225 Hz, and the amplitude *g*_*L*_ corresponds to either 30 dB (Fig 6A) or 90 dB SPL. In these simulations, to account for the nonlinearities of the cochlea in the case of the higher sound level, we used the more updated model of Zilany et al. [27–29]. Fig 6A shows that for the 30 dB SPL signal, the AN population response is limited to a small region around the CFs of the stimulus’ spectral components, as expected. On the other hand, for the 90 dB SPL case (Fig 6B), the AN population response is dramatically different as it spreads out along the whole cochlea. Moreover, since the response is heavily saturated, the spatiotemporal patterns of peaks and troughs for each of the AN fibers are biased relative to the case of the moderate sound levels. Despite this significant difference in the AN responses between different sound levels, psychoacoustic measurements indicate that the sensation of pitch is robust to that effect [6,44].

The output of the SC model, **h**, is shown in Fig 6C and 6D for the two stimuli levels, 30 and 90 dB-SPL, respectively. Each panel shows both the sparse (SC, λ = 0.01, red) and non-sparse (LS, λ = 0.0, blue) solutions. As can be expected, the number of nonzero components in the vector **h** is much smaller for the SC solution when compared with that obtained by the LS algorithm. This sparseness applies to both sound levels, but the difference between the two solutions is much more noticeable for higher sound level (compare the blue line in Fig 6C with that of Fig 6D). Specifically, the LS solutions for the two stimuli levels are fundamentally different, and there is no apparent preservation of the spectral components of the input stimulus. Thus, the LS solution is variant to sound level, as opposed to what we would have expected from a representation of a pitch in the auditory system [45]. In comparison, the SC solutions do manage to preserve their overall structure. While the two SC solutions are not identical, both have only a few non-zero terms that directly relate to the frequency components of the input stimulus. Consequently, it seems that the sparse requirement in Eq 2, at the expense of the accuracy of the ordinary LS solution, contributes to the invariant representation of the stimulus in the vector **h**, regardless of its sound level.

In order to understand the effect of these different representations on pitch perception, we compare the resulting probability density functions (the *pdf*(*f*_*p*_)) for the two stimulus levels and both LS and SC solutions (Fig 6E and 6F). For the low sound level (Fig 6E and 6F), the two solutions are alike, and there is no apparent benefit to using one over the other: both curves (blue and red) have peaks at the same frequencies, and the maximum probability point equals that of the stimulus' fundamental frequency, i.e., ${\hat{f}}_{0}=225\;\text{Hz}$. The result is substantially different for the 90 dB SPL amplitude. In this case, the pdf that corresponds to the solution of the LS algorithm has lost all resemblance with the stimulus' frequency components—it is just a flat, noisy curve. In comparison, the pdf that corresponds to the SC solution still has a clear indication of the original stimulus properties. In particular, the pdf curve (red line in Fig 6F) peaks at the harmonics of the fundamental f_0_, with a maximum peak at ${\hat{f}}_{0}=225\;\text{Hz}$.

### Effect of Different Dictionaries

Unlike, for example, the Fourier transform, the SC transform enables the use of various dictionaries that can be set according to some desired specifications. A standard option is to train a dictionary according to desired optimal constraints [35], but this is certainly not a prerequisite. For instance, when mapping patches of images into their parsimonious representations, one can choose to set the atoms of the dictionary as the basis of the discrete cosine transform, which is a straightforward and efficient choice (see for example Ch.12 in [25]).

In the current study, we checked two families of dictionaries: the first (*D*_sine_) contains atoms created by sine stimuli, and the second (*D*_*stack*_) contains atoms created from harmonics tones stimuli (harmonic stack). Specifically, each of the atoms in *D*_sine_ was produced by stimuli of one tone with random (uniformly distributed) amplitudes, and the atoms of *D*_*stack*_ were created by complex tone stimuli (harmonics 1^st^-6^th^) of the same moderate amplitude level (see Methods)

**Fig 7:**
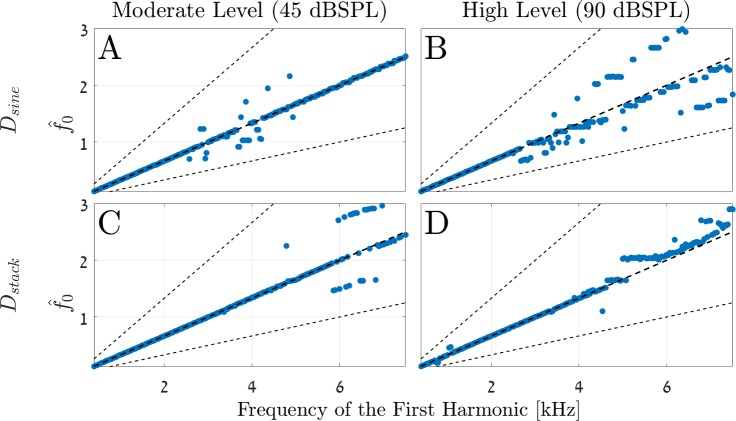
Comparing the performance of different dictionaries over moderate and high amplitude stimulus levels. All simulations have the same spectral structure (Eq 6). This spectral structure is simulated for various fundamental frequencies, *f*_0_, and the figures show the estimated pitches for each such case (i.e., the maximum peak in each pdf). The estimations are taken from an interval of ± 0 .5 octaves around *f*_0_. Each row, i.e., figures A-B and figures C-D, show the estimation results of the SC model for the two dictionaries *D*_sine_, and *D*_*stack*_, respectively (see text). The column subplots refer to different stimuli levels: moderate (45dB SPL), and high (90dB SPL) amplitudes. The x-axis denotes the location of the first harmonic within the stimuli (i.e., the 3^rd^ harmonic); the thick black dashed lines define the main octave (*f*_0_), and the thin black dashed lines define the lower and upper octaves, i.e., 0.5 *f*_0_ and 2*f*_0_, respectively. (A-B) At low frequencies, up to about 4k Hz of the lower harmonic in the complex stimulus, the estimations of the *D*_sine_ dictionary converge to the expected frequencies for both moderate and high stimuli. However, from 4k Hz and above, the pitch estimations for the high stimuli levels diverge from the main octave to other ratios of *f*_0_. (C-D) The pitch estimations of the *D*_*stack*_ dictionary converge to the main octave better for the low and high frequencies and for both amplitudes.

The two dictionaries were checked with both stimuli of 45dB SPL (Fig 7A–7C) and high amplitude levels of 90dB SPL (Fig 7B–7D). All simulations had the same spectral structure as given by Eq 6, i.e., all signals were complex tones with the 3–8 harmonics (the missing fundamental case). The results show the maximum peaks (blue dots) of the resulting pdfs that are taken from an interval of an octave around the fundamental frequency, as is the practice in psychoacoustic measurements (see for example [41,42]).

Comparing the four subplots of Fig 7, the dissimilarities between the results of the two dictionaries demonstrate quantitatively different results but qualitatively similar performances, at least in the lower frequency regions. For example, the two dictionaries yield relatively good estimations of the pitches up to about 4k Hz of the first stimulus’ harmonic (the third harmonic). For frequencies higher than 4k Hz, there is more variance around the expected fundamental frequency (thick black dashed middle line). Specifically, the 90dB SPL estimations for both *D*_sine_ and *D*_*stack*_ seem to be horizontally spread (Fig 7B–7D). From our experience, these deviations can be reduced by using dictionaries of higher resolution (i.e., with more cochlear channels and more atoms). However, due to the naïve structure of this model, there is a computational limit to the dictionary size that we can use. This restriction will hopefully be alleviated by a future model (see Discussion).

**Fig 8:**
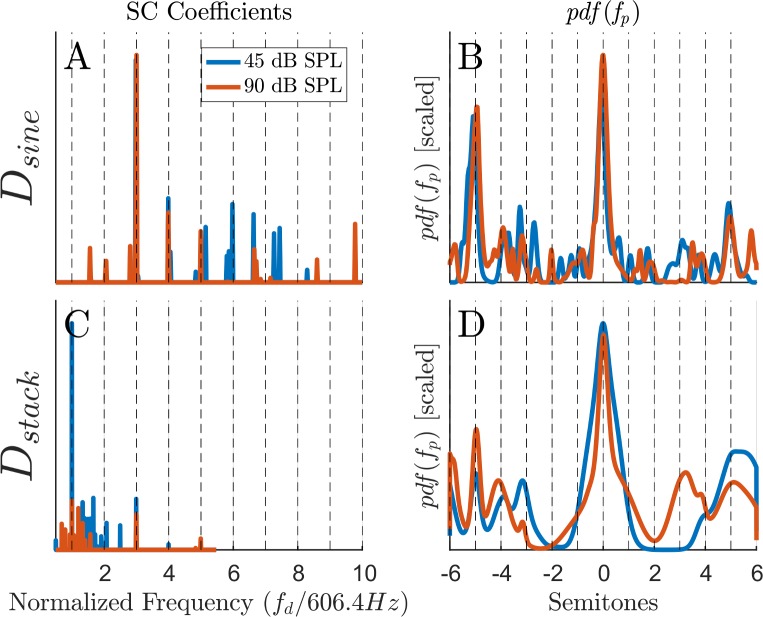
Detailed results for *f*_0_ = 606.4*Hz*. The selected examples are taken from Fig 7 and show the SC coefficient vectors **h** and the pdfs for the two dictionaries and for the two amplitudes. **(A, C)** The SC coefficient vectors **h** for the *D*_sine_ and *D*_*stack*_ dictionaries, respectively. **(B, D)** The resulting pdfs, over one octave around *f*_0_ = 606.4*Hz*, for the *D*_sine_ and *D*_*stack*_ dictionaries, respectively. Note the difference between the SC coefficients of the two dictionaries, but the qualitative resemblance between the two pdfs.

Fig **8** shows in more detail four selected examples of *f*_0_ = 606.4 *Hz*. The two **h** vectors in Fig **8**A are taken from the moderate and high amplitudes levels of the *D*_sine_ dictionary case. The performance of the model for the 90dB level is a bit degraded compared to the 45dB level, as expected (see also Fig 6). Specifically, in the 90 dB SPL, the model estimates lower coefficients in **h** due to the nonlinearity of the cochlea. The resulting pdf, i.e., the probability for a particular pitch given these SC coefficients, is shown in Fig **8**B. Fig **8**C and **8**D show the **h** vectors and pdfs for the second dictionary, the *D*_*stack*_. Comparing Fig **8**A with Fig **8**C for the respected amplitude levels shows that the two SC vectors have different coefficients. This difference is due to the particular structure of the atoms in each of the two dictionaries above. Still, in spite of this structural differences, the **h** vectors have nonzero coefficients over indices that represent harmonics of f_0_. Consequently, the resulting pdfs have qualitatively similar results and the maximum peaks appear at the same location. Thus, the result is that the SC model predicts the same pitches for these two cases. Interestingly, the harmonic sieve is designed, in principle, to be optimal in the case of *D*_sine_ (and under certain assumptions, see [19,40]). Even so, it can still be used for the *D*_*stack*_ dictionary and yield good results. Note that it might be that for the *D*_*stack*_ dictionary there is a better (in the aforementioned optimal sense) representation for the harmonic sieve, but we did not pursue this path any further.

In summary, this section emphasizes the notion that choosing a dictionary can improve or reduce the performance of the model in different aspects. Thus, one emerging interesting question from the above discussion is which dictionary can be acquired in a biologically compelling manner to match psychoacoustic and physiological measurements best? In this paper, however, we do not address this issue, but we have chosen to focus on demonstrating that parsimonious representation of an auditory information can explain relatively high cognitive tasks, i.e., the percept of a pitch. In what follows we assume a dictionary that is built of a single tone. Hopefully, the choosing of such a relatively simple dictionary, instead of a more intricate one, would prove to be clear and emphasize the qualitative abilities of such an approach.

### Resolved and Unresolved Harmonics

The harmonics of a periodic signal are spatially distributed along the BM. Because of BM properties, low harmonics create separate peaks that are translated into distinct excitation patterns in the activities of the ANs. Since higher harmonics, on the other hand, do not yield such distinct peaks, these harmonics do not have distinct excitation patterns. Consequently, low harmonics are referred to as resolved and higher harmonics, approximately at the 5^th^–10^th^ harmonics [14], as unresolved. For unresolved stimuli, the temporal aspects of AN response convey more information about the pitch than the spatial aspects of that response. Thus, using stimuli with resolved and unresolved harmonics is a controlled way to inspect the temporal processing of a pitch in the auditory system.

Broadly speaking, temporal models, such as the summary autocorrelation function (SACF) [4], disregard the resolvability of the stimulus. This is because such models account for the interactions between the harmonic components of the periodic signal whether they are resolved or not [44]. This indifference, however, stands in contrast to psychoacoustic observations [11,12,14,46].

**Fig 9:**
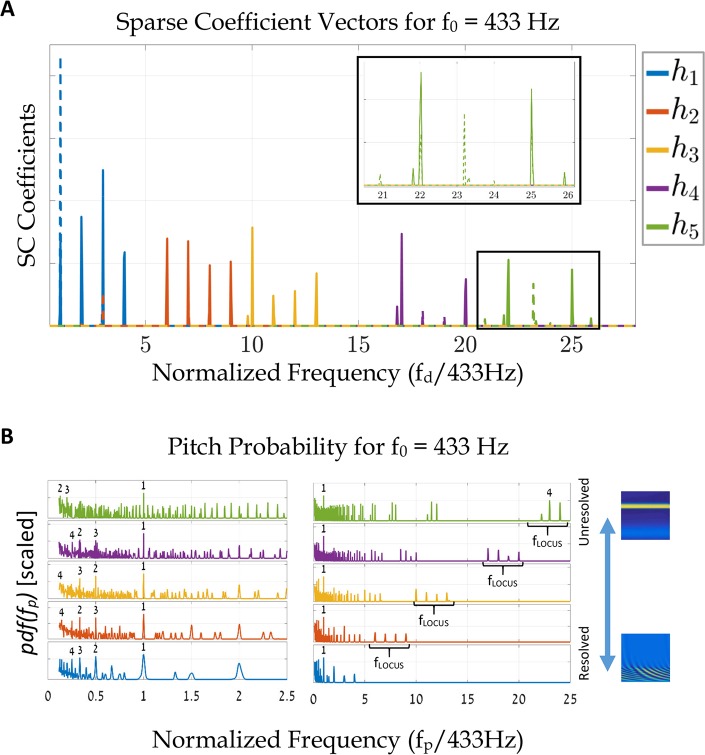
Resolved vs. unresolved representation of harmonic cues. **(A)** The solutions **h**_k_, k ∈ [1, 5], for the stimuli of Eq 7. We compare the SCs of the two dictionaries, *D*_sine_ (lines) and *D*_*stack*_ (dashed lines). *D*_sine_ consists of tone-atoms and *D*_*stack*_ consists of complex tones that contain six harmonics with decreasing amplitudes (1 to 1/6). All stimuli contain four harmonics of the same fundamental frequency, f_0_ = 433 Hz, but at different spectral locations (r ∈ {1, 6, 10, 17, 22}). The x-axis is normalized by f_0_ for convenience. The correlation between the SC solutions and the stimuli' spectral components (Eq 7) are apparent. Note that signals with low-frequency components (such as **h**_1_) have more prominent nonzero coefficients than those of the higher harmonics (e.g., **h**_5_). A closer look at **h**_5_ (the inset) shows that only two of the four harmonics are successfully reconstructed (the 23 and 24 tones of the 22–25 harmonics). **(B)** Pitch probabilities (pdfs) for the five complex tones for the *D*_sine_ (see text). The right figure shows all f_p_ frequencies and the left one views fewer octaves around f_0_. The numbers above the curves state the four prominent peaks of the pdfs, from the highest (1) to the fourth lower peak. Observe that all five solutions peak at the first harmonic, that is, the model predicts the same 433 Hz pitch for all stimuli. Additionally, most of the other plausible pitches, i.e., other peaks, are usually located at harmonic ratios of f_0_, that is, they represent octave equivalence options. It is also instructive to note the f_LOCUS_ frequencies in the right figure of (B). These peaks indicate the additional possibility of perceiving the pitches at the locus of the stimuli spectral energy and not of f_0_ [1]. All simulations were performed with Slaney's model and with a sound level of 45 dB SPL.

The current model combines temporal and spatial aspects of the AN response within its atoms. Thus, it is interesting to examine the model’s response to this class of stimuli. We compare five stimuli of complex tones (Fig 9),

$$s_{in}\left(t\right)=g_{45dB}\cdot \left(\sum_{k=r}^{r+3}\sin\left({2\pi \text{f}}_{0}\text{k}\cdot \text{t}\right)\right).$$ (7)

In Eq 7, each stimulus has the same spectral structure, that is, four consecutive harmonics, but the spectral locations of harmonics vary. Specifically, the spectral location of the first harmonic in each signal is set by r ∈ {1, 6, 10, 17, 22}. Additionally, the fundamental frequency is configured to f_0_ = 443 Hz, and the gains (*g*_45 *dB*_) of all stimuli are equivalent to 45 dB SPL.

Fig 9A shows the sparse coefficient vectors **h** for all five stimuli. Additionally, we compare the SCs of the two dictionaries, *D*_sine_ (lines) and *D*_*stack*_ (dashed lines). *D*_sine_ consists of tone-atoms and *D*_*stack*_ consists of complex tones that contain six harmonics with decreasing amplitudes (1 to 1/6). All the atoms are created with 60 dB SPL stimuli. As can be seen, there is a slight difference between the results, but overall both dictionaries yield the same SCs, pdfs, and pitch estimations. For that reason, we focused the rest of the analysis on the *D*_sine_ results.

In Eq 7, Low values of r represent stimuli with resolved harmonics while larger values (r > 5) represents stimuli with unresolved harmonics. Observe (Fig 9A) that for most of the stimuli, the four prominent spectral components of **h** are successfully reconstructed. Still, there is an apparent degradation (from resolved to unresolved stimuli) in the amplitude of the coefficient terms. For resolved stimuli, e.g., the 1^st^–4^th^ harmonics stimulus (r = 1), the **h** vector holds prominent terms equivalent to the frequency components of the input signal. As r increases, the estimated terms in **h** decrease; for example, in the 22^nd^–25^th^ harmonics stimulus (r = 22), the vector **h** only contains two out of the four equivalent frequency components of the input signal (Fig 9A, inset). This degradation is due to the reduced ability of the AN population to phase-lock with high frequency stimuli. Thus, the match between the atoms and the stimuli is less accurate, which results in smaller SC coefficients.

Fig 9B shows the corresponding pdfs of each of the five stimuli. The right panel shows the entire pdf while the left panel focuses on the vicinity of the fundamental f_0_ = 443 Hz. The numbers in the figure indicate the arrangement in a descending order of the peak heights, starting from the highest peak (peak number 1). For all stimuli, the maximum of each density function was obtained at the fundamental frequency. It is instructive to note that for each of the stimuli, other local maxima, indicating other pitch possibilities, are allocated at harmonic ratios of f_0_. For example, in the case of the lowest harmonic complex (r = 1), maximum peaks in the pdf are also available at the f_0_/4, f_0_/2, and 2f_0_. These other options represent the octave equivalence of the perceived pitch. Usually, humans perceive these options to be the same pitch, or to have the same pitch chroma. Hence, in psychoacoustic measurements, this mixing between octaves is generally not considered as an error [41–43] (specifically, see SI in [41]).

**Fig 10:**
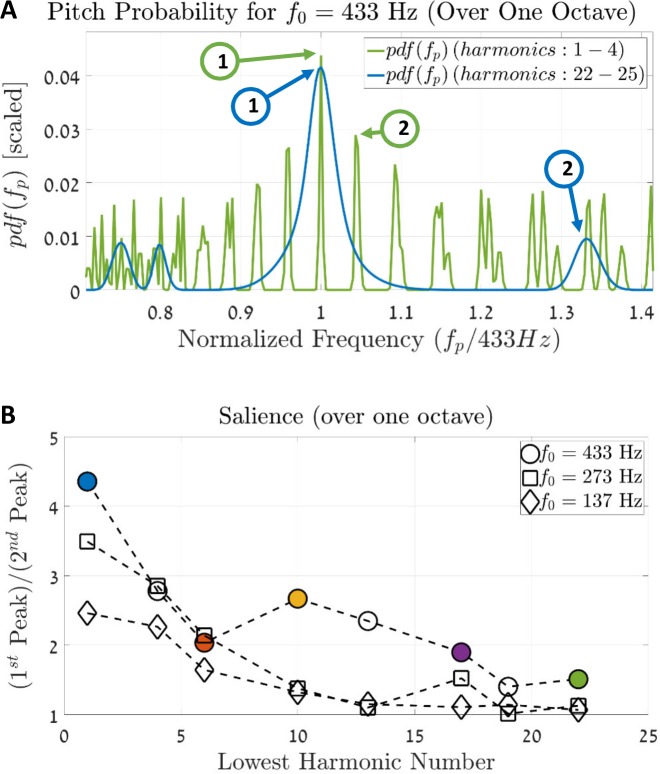
Salience of complex tones. **(A)** A Comparison between the two probability functions of the complex tones from Fig 9: the blue line is the pdf of the complex harmonic tone with the 1–4 harmonics, and the green line is the pdf of the fifth stimulus, which comprises 22–25 harmonics. The x-axis is limited to one octave in order to compare the pitch's relative heights and without considering the octave equivalence of consecutive harmonics. The blue and green arrows show the 1^st^ and the 2^nd^ largest peaks of the two curves, respectively. Computing the ratio for each curve between the 1^st^ and the 2^nd^ peaks yields a measure of the pitch's salience; a larger ratio indicates a more prominent percept of tha pitch. **(B)** Calculating the ratio between the 1^st^ and 2^nd^ peaks for harmonic tones with four consecutive tones at different harmonic numbers. The x-axis indicates the location of the first harmonic in each stimulus, and the y-axis shows the ratio between the 1^st^ and the 2^nd^ peaks (as demonstrated in (A)). Colored circles indicate the relevant stimuli that are shown in **Fig 9**.

In Fig 10 we compare the pdfs of the two complex tones of Fig 9. The stimulus that contains the 1–4 harmonics is shown in blue while the stimulus that contains the 22–25 harmonics is in green. This comparison is done over one octave to avoid the other pitch equivalence solutions (which are approximately at the same height of the 1^st^ peak). By inspection (Fig 10A), the ratio between the 1^st^ and the 2^nd^ peaks is higher for the resolved stimulus (blue line) compared to that of the unresolved stimulus (green line). We chose to denote this difference in the ratios as the salience of the stimuli. Fig 10B shows a comparison of additional stimuli with different fundamental frequencies; each one is a complex tone that contains four consecutive tones. The colors of the circles match the stimuli colors of Fig 9. Note that the salience of all stimuli decline with the increase of the location of the first harmonic, i.e., as the harmonics transcend from resolved to unresolved. Finally, stimuli with high harmonics also have additional peaks around the locus of the harmonic components (see the f_LOCUS_ peaks in Fig 9B). These phenomena are consistent with known physiological data [9].

To conclude, despite the model’s seemingly spatially-based nature, it can derive the pitch of unresolved harmonics to some extent. Unlike purely temporal models, however, it penalizes these stimuli in relation to resolved ones. Note that this penalty is a consequence of the cochlear properties and not of the SC module. Specifically, this penalty was not introduced artificially into the model—it is an implicit property of the atoms and stems directly from the properties of the cochlear model.

### Pitch Shift of Inharmonic Equally Spaced Tones

Perceived pitches are usually considered within the context of periodic signals. For example, the perceived pitch of a complex tone is its fundamental frequency, f_0_, whether it exists in the complex or not. Consider the following harmonic series

$$s_{in}\left(t\right)=g\cdot \left(\sum_{k\in \left[k_{0},k_{1}\right]}\sin\left(2\pi \left({\text{f}}_{0}\text{k}+\Delta \text{f}\right)\cdot t\right)\right),$$ (8)

For Δf = 0, human subjects usually perceive the pitch of s_in_(t) as f_0_, the fundamental frequency of the harmonic signal [47]. This is true even for cases when f_0_ is not present in the signal, i.e., k_0_ > 1. For increased Δf > 0, the expression in Eq 8 is no longer harmonic. Nonetheless, previous psychoacoustic experiments have revealed that human subjects do manage to perceive pitches with these shifted stimuli, and the detected pitches are approximately shifted on a linear scale relative to the fundamental frequency [9,40,47–49]. These kind of stimuli are important because they demonstrate that pitch detection does not follow the stimulus' envelope, which does not change in this case (this is not true for stimuli with unresolved harmonics); nor does it follow the spacing between frequency components of a stimulus [1]. Thus, the phenomenon of pitch shift was used as a counter example for models that exploit the temporal envelope of a stimulus or other of its temporal features, such as zero crossing, peaks, etc. [18].

**Fig 11:**
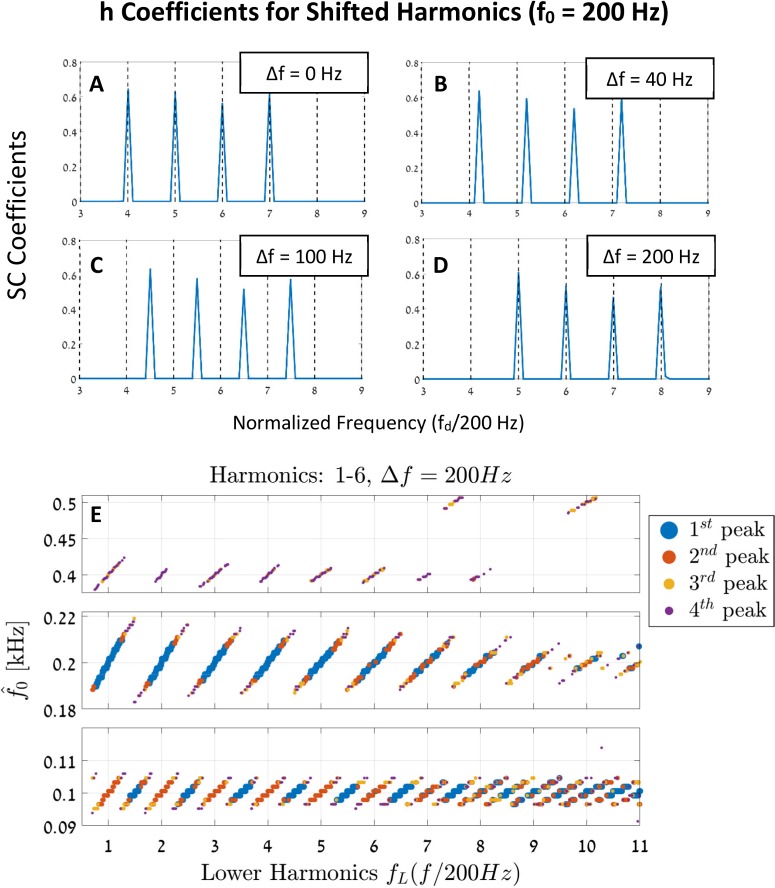
Pitch shift of equally spaced harmonics. **(A-D)** The vectors **h** for complex harmonic stimuli that contain the four harmonics of 4–7 (Eq 8). The x-axis denotes f_d_ normalized by the fundamental frequency, f_0_ = 200Hz. The four figures show the stimulus in Eq 8 for the cases of Δf = 0 Hz, 40 Hz, 100 Hz, and 200 Hz, respectively. The zero shift case represents a regular complex harmonic signal. The 40 Hz shift is no longer a complex tone of 200 Hz. The third option (C) is a harmonic complex of 100 Hz (with the harmonics 9, 11, 13, and 15). Finally, the Δf = 200 Hz shift results again in a complex harmonics of f_0_ = 200 Hz but this time with the 5–8 harmonics. **(E)** The peaks of the probability functions, *pdf*(*f*_*p*_), for 500 uniformly shifted stimuli. Each stimulus is given by Eq 8, i.e., each signal includes the first four terms (1–4) of the fundamental f_0_ = 200Hz, plus an incremental frequency shift of Δf. The x-axis denotes the frequency of the lowest harmonic component of the input stimulus (f_0_ + Δf) normalized by f_0_ for visual clarity. The y-axis denotes the estimated pitch. To demonstrate the ambiguity of this process, we included the first four largest peaks of each of the resulted pdfs. We focused the view along the 100 Hz, 200 Hz, and 400 Hz in the y-axis; all other regions are mostly empty. Note the linear shifts in the pitch estimations and the changing of these slopes as a function of Δf [47].

Fig 11A–11D shows the model's solutions of the sparse coefficient vectors, **h**, for different shifted signals (Eq 8). In these simulations, the input signal, s_in_(t), has four frequency components that are set to k ∈ [4,7], and the (missing) fundamental frequency is set to f_0_ = 200 Hz. In this example, setting Δf = 0 creates a stimulus s_in_(t) that is a complex harmonic series of f_0_ = 200 Hz. For a shift of Δf = 40 Hz, the stimulus is no longer a complex tone of f_0_ = 200 Hz, and a shift of Δf = 100 Hz changes the stimulus to be a complex tone of f_0_ = 100 Hz (with the [9, 11, 13, 15] harmonics). Finally, for a frequency shift of Δf = 200 Hz, the input signal is once again a complex tone of the fundamental f_0_ = 200 Hz, but this time with the [5, 6, 7, 8] harmonics. Following these observations, we would expect the model to exhibit this ambiguity by the frequency shift Δf, and to alternate its predictions between the frequencies of f_0_, 0.5f_0_, 2f_0_, etc.

We performed 500 simulations of s_in_(t) (Eq 8) with f_0_ = 200 Hz. Each signal contains the first six harmonics (i.e., k ∈ [1, 6]), and each is simulated with a different Δf. Fig 11E shows the estimated frequency ${\hat{f}}_{0}$ as a function of the lower harmonic component *f*_*L*_ in the input signal. For each of the stimuli, the corresponding pdfs are calculated, and the four highest peaks are indicated. We chose to include the four prominent peaks of each pdf to show the ambiguities of these signals. The estimated pitches are clustered in lines as a function of the lower frequency *f*_*L*_, consistent with the known “first effect of pitch shift” [47]. One can also see that as the shift Δf increases, the slopes of the estimated lines slightly decrease, in accordance with the known psychoacoustic phenomenon [47].

To conclude, the current model qualitatively reproduces the known psychoacoustic phenomenon of pitch shift, even though these aperiodic signals are not part of the model's dictionary. This implies that the current model: generalizes to complex new stimuli; it does not depend on the stimulus temporal envelope for cues; and it does not use the spacing between the stimulus harmonics to estimate pitch.

### Transposed Tones

Transposed tones (TTs) were first introduced to explore the relative sensitivity of the auditory system for binaural timing stimuli [50]. Oxenham et al. [13] used these signals to check the relation between the tonotopic organization in the cochlea and the perception of pitch. The motivation of using these signals is to introduce low-frequency temporal structures into the basal part of the cochlea that usually processes high frequencies. TTs are produced by the modulation of half-wave rectified sine waves with carrier waves. Due to the limited synchronization and low-pass properties of the basilar membrane, the outer hair cells, and the ANs, the fine details of the carrier waves would be negligible. Thus, the results are half-wave rectified sine waves of low frequency (f_0_) in high CFs regions (> 4 kHz). In this manner, there is a separation between spatial locations and fine temporal structures along the cochlea.

Measurements carried out by Oxenham et al. [13] showed that subjects could not estimate the fundamental frequency of the TT stimuli. This inability means that the spatial arrangement of CFs along the cochlea is essential for the perception of pitch. Oxenham et al. have also shown that the summary autocorrelation function (SACF), a well-known temporal model, is indifferent to TTs and thus to the tonotopic organization in the cochlea. When applying TTs to the analysis of the SACF, the model does manage to extract the correct fundamental frequencies, in contrast to the aforementioned psychoacoustic evidence.

**Fig 12:**
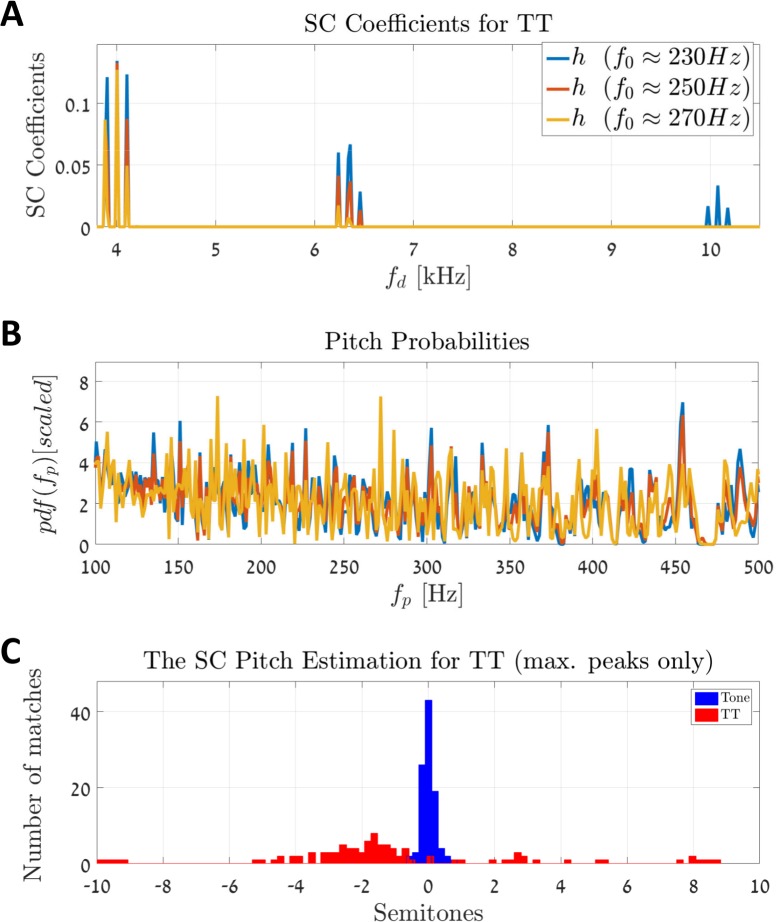
Transposing low-frequency tones into high-frequency regions of the cochlea. **(A)** An example of three sparse coefficient vectors, **h**, for the three frequencies f_0_ = 229 Hz, 249 Hz, and 269.7 Hz. The resulting **h** vectors have the same nonzero indices, i.e., these stimuli cannot be differentiated based on their sparse representations. **(B)** The pdfs of the three TTs are noisy and inconclusive, as expected. **(C)** Predictions of 100 epochs; only the 1^st^ peak in the pdf is considered. There are two distinct types of stimuli: (i) pure tones (blue), and (ii) TTs (red). Both stimuli are simulated with incremental fundamental frequencies of f_0_ ∈ [100 Hz, 500 Hz]. Each stimulus is normalized relative to the fundamental f_0_. The model could estimate the f_0_ of the pure tones with a high degree of accuracy but could not predict those of the TTs at all (compare with [13]).

In the case of the proposed model, each region along the cochlea is characterized by its local spatiotemporal activities. These localized patterns are embedded in the atoms for each CF. Hence, we predicted that the SC model would not be able to separate between spatial and temporal processing. Fig 12 shows the processing of TT stimuli by the SC model. We first considered three TTs with the fundamentals f_0_ = 230 Hz, 250 Hz, and 270 Hz. The expression for each of these TTs is given by

$$s_{TT}\left(t\right)=g_{30dB}\cdot \sum_{k\in \left[1,3\right]}{\bar{s}}_{k}\left(t\right)\cdot \sin\left({2\pi \text{f}}_{\text{c},\text{k}}^{}\cdot t\right).$$ (9)

In this equation, ${\bar{s}}_{k}\left(t\right)$ is a low-pass filtered version of a rectified sine wave (see Methods), and each of these tones is modulated by the three carriers: f_c,1_ = 4 kHz, f_c,2_ = 6.35 kHz, and f_c,3_ = 10.08 kHz. Fig 12A shows the results of the sparse coefficient vectors (**h**) for the above three TT stimuli. It is apparent that the three sparse representations occupy the same indices, i.e., the same frequencies f_d_ in **h**. Therefore, the SC model cannot distinguish between these stimuli based on their sparse representations. And indeed, the pdfs of these sparse vectors are inconclusive (Fig 12B).

Next, we simulated a batch of 100 epochs (Fig 12C). Each epoch contained the TT of Eq 9, and each had an incremented fundamental frequency taken from the interval f_0_ ∈ [100 Hz, 500 Hz] (red bars in Fig 12C). We repeated the simulation also for pure tones (blue bars in Fig 12C) and compared the two by normalizing the measurements with the respective f_0_. The results are consistent with the findings of Oxenham et al., that is, the SC model could not estimate the f_0_ for the TT stimuli successfully.

### Iterated Rippled Noise

Delaying a signal of broadband white noise and adding it back to the original one creates a signal known as rippled noise. When this process of delaying and adding is repeated, a signal known as iterated rippled noise (IRN) is created. These signals contain temporal regularities in the time domain and spectral peaks at the reciprocal of the delayed time in the spectrum domain. Due to the nature of these signals, human listeners report perceiving two sensations: a tonal part that amounts to the pitch of the reciprocal of the delay (*d* ms) and an additional noisy sensation [51,52]. Repeating the iteration process results in a more prominent sensation of the tonal pitch [53].

Adding the delayed noisy signal back to the original one with a gain of one (delay-add) yields a signal with spectral peaks that are located at the reciprocal of the delay time *d*. But adding the delayed signal with a gain of minus one (delay-subtract) yields a signal with peaks in the power spectrum that are shifted by 1/2*d*, as if the delay-subtract signal is an odd-harmonic complex of half the frequency of the delay-add version. Delay-add stimulus raises a sensation of pitches of 1/*d* Hz, whereas delay-subtract is usually perceived to be more ambiguous and yields pitches that are slightly higher or lower than 1/*d* Hz [53].

**Fig 13:**
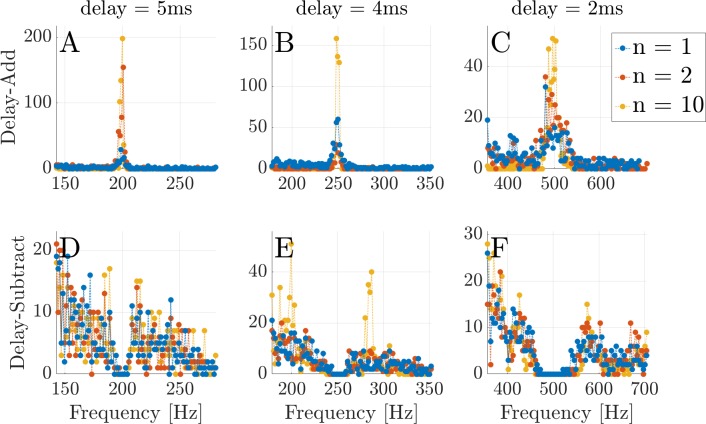
Iterated rippled noise for different time delays and repetitions. The figures show the results of 500 simulations for each case of IRN stimulus. Each subplot along the columns show the delays of d = 5, 4, and 2 ms that correspond to the fundamental frequencies of 200, 250, and 500 Hz, respectively. The subplots in the first row show the delay-add simulations, and the lower row shows the delay-subtract simulations. The results are derived from the first peaks of the resulting pdfs, and all estimations are taken from an interval of one octave around the appropriate fundamental frequency [42]. Simulations are done using Carney's model (Zilany et al. [27–29]) with stimuli of 70 dB SPL. The dictionary contained 1000 groups of sine-atoms with distinct CFs and 10 phases in each group (g = 10, Eq 3).The blue dots indicate rippled noise (one repetition), red points correspond to IRN with 2 repetitions, and yellow dots are for the 10 repetitions. **(A-C)** The delay-add simulations show distinct peaks around the 1/*d* frequencies. **(D-F)** The delay-subtract simulations show accumulation of the inferred pitches at frequencies equal to or greater than 1/*d*±10%, but the results for this case are noisy and inaccurate relative to psychoacoustic measurements.

Simulations of delay-add and delay-subtract stimuli with the delays of d = 2, 4, and 5 ms, are shown in Fig 13. These signals are created as follows: for a white noise, *x*(*t*), the iterated signal *s*_*n*_(*t*) is created by

$$\begin{array}{l} s_{i}\left(t\right)=s_{i-1}\left(t\right)+g\cdot s_{i-1}\left(t-d\right)\\ s_{0}\left(t\right)=x\left(t\right) \end{array},$$ (10)

for *i* = 1,…,n_*itr*_, and n_*itr*_ is the number of iterations (e.g., 1, 2, or 10). All simulations were done using Carney's model (Zilany et al. [27–29]) with stimuli levels of 70dB SPL; the dictionary contained 1000 sine-atom groups and each group has 10 time-shifts (phases) in it (g = 10, Eq 3).

In the first row (Fig 13A–13C), which contains the delay-add cases, a clear peak appears at the reciprocal of the delay, 1/*d*, as expected. As the number of repetitions increases, so does the prediction quality of the model, i.e., more estimations are concentrated around 1/*d* Hz. In Fig 13D–13F we show the delay-subtract cases for the same delays. In these simulations (Fig 13E and 13F), the inferred pitches are located around the reciprocal of the delays, as expected. But we would also expect the measurements to peak at approximately 1/*d*±10%, which does not happen.

### Musical Notes

Music in the Western culture is based on a musical scale that relates periodic (or quasiperiodic) sounds to their fundamental frequencies. Thus, musical instruments that are based on this musical scale produce harmonic sounds based on these fundamental frequencies. As such, different instruments have different spectral coloring (i.e., timbre), but human listeners can perceive and compare the fundamental frequencies between the instruments [54]. This ability is due to the pitch perception property of clustering periodic (or quasiperiodic) sounds into classes, i.e., musical notes.

**Fig 14:**
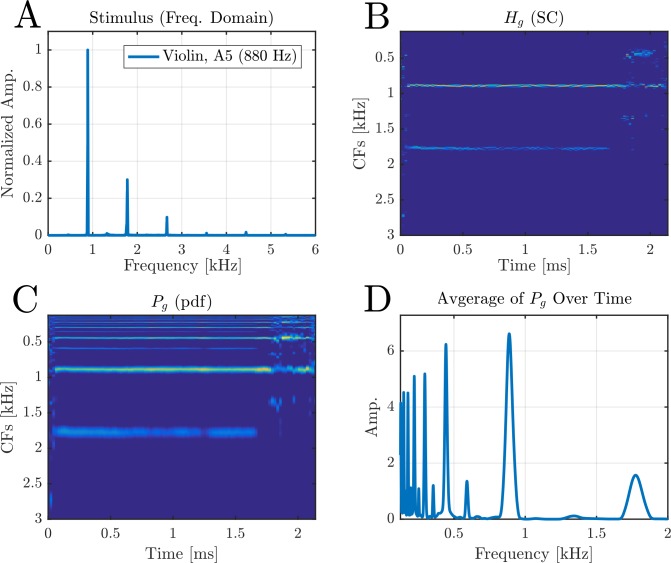
Analyzing a recorded stimulus of a violin. **(A)** The Fourier transform of the recorded signal. This is a note of A5 (880 Hz) played by a bow (arco). The 880 Hz and its harmonics are clearly seen. **(B)** Each time step *T*_*steps*_ of the stimulus is processed separately. The results are collected to form the columns of the matricx *H*_*g*_. **(C)** Each of the SC vectors (columns) of *H*_*g*_ are processed by the harmonic sieve separately to produce the pitch probability of that time step (*P*_*g*_). **(D)** To compare between simulations, we average over the time steps to extract the most prominent pitch of the signal. The result is the usual pdf vector, and the estimated pitch is set to the maximum of this pdf.

In this section, we checked the SC model with recorded musical notes [55]. For this, we used a dictionary with 1000 atoms, each of which had ten different phases (g = 10, Eq 3). Each recorded stimulus was divided into *T*_*steps*_ = 100 time steps that were analyzed separately. Fig 14A shows the FT of a recorded violin note of A5 (880 Hz) played with a bow (arco). At each time step *T*_*steps*_, Eq 3 is solved separately to obtain $\tilde{h}\in R^{gM}$. Next, the coefficients of each group are summed together to get the SC vector **h** ∈ *R*^*M*^. The matrix $H_{g}\in R^{M\times T_{steps}}$ is the aggregation of all **h** ∈ *R*^*M*^ over *T*_*steps*_ = 100 steps (Fig 14B). Each of the columns of *H*_*g*_ (the SC vectors) are then processed by the harmonic sieve unit to produce the probability of that time step. The collection of all these pdf is given by the matrix $P_{g}\in R^{M\times T_{steps}}$ (Fig 14C). Finally, to have one single probability for each stimulus, the matrix *P*_*g*_ is averaged over the time domain and normalized appropriately (Fig 14D). As in previous cases, the pitch of the signal is defined as the maximum point in this pdf.

**Fig 15:**
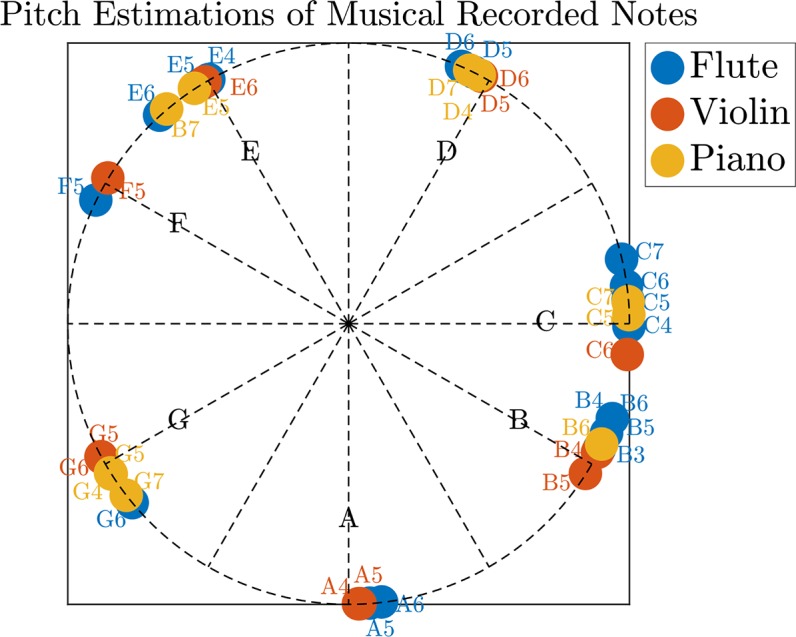
Results for musical notes on a chromatic scale. We analyzed three musical instruments: a flute, a violin, and a piano for different notes. The results are shown on a chromatic musical scale (equal-tempered). The colored labels along the colored dots specify the notes played in specific recordings. All of the recordings were downloaded from [55]. Although not exact, the model does manage to assign most of the measurements to the right note (pitch).

We repeated this procedure with recorded notes of a flute, a violin, and a piano (Fig 15). All results are shown on a chromatic scale. Each dot in Fig 15 is the estimated pitch of a recorded instrument; the colored text indicates the played note.

## Discussion

We showed that a model based on the sparse coding of the spatiotemporal pattern of auditory nerve responses is consistent with many pitch perception phenomena. The model represents input stimuli as sparse linear combinations of atoms, where each atom is derived from the AN population response to a pure tone.

Since the perception of pitch can be elicited by a variety of different stimuli [31], we tested the model on various such categories. We demonstrated that the sparse representation arising from a given stimulus at different sound levels could be linked to the spectral components of that stimulus, giving rise to a level-invariant representation of a pitch. Resolved and unresolved stimuli lead to a different pitch estimate in the model, with the difference stemming directly from cochlear properties. Inharmonic stimuli were used to show that the model can generalize to new stimuli while relying neither on the spacing between harmonics nor the temporal envelope of the stimulus. Next, we demonstrate that the use of the ANs spatiotemporal patterns as atoms force a tonotopic structure into the model. Consequently, it cannot estimate transposed tones (TTs), in accordance with known psychoacoustic measurements [13]. We showed that the model complies with IRN stimuli, and it is also able to process the recorded sounds of musical notes.

The focus of this paper was the application of sparse coding to the problem of pitch perception. The particular choice of the supporting elements used here (i.e., cochlear model, pitch estimation unit, and LASSO) are somewhat arbitrary. First, we chose standard, biologically inspired, cochlear models [26–29]. Second, we implemented the sparse coding (SC) algorithm by a known algorithm with available implementations, the LASSO [32]. Other plausible choices are presented in the literature, such as matching pursuit algorithms [25,56] (see [57] for different implementation in the auditory system). Third, the final pitch-estimation phase was implemented as an instance of the commonly used [58] harmonic sieve (via pattern-matching models). This construction enables an algorithm-level view [59] of the topic at hand.

### The SC Model and Related Models for Pitch

The SC model presented here combines both spatial and temporal aspects of the AN population response. On the one hand, the SC model is based on atoms that have limited spatial support, namely, the nonzero section along the BM that is given by the equivalent rectangular bandwidth of the cochlea [60]. On the other hand, each atom also includes temporal information about the activities of the AN fibers at that spatial location.

The current model is structured in a similar manner to pattern-matching models [5,16,18]. The Fourier-like spectrum analyzer that extracts the resolved harmonic components of a given stimulus [18] is modified to include both the cochlea and the SC modules. Both models use templates to associate an estimated pitch with presented stimuli [58,61]. Despite these similarities, there is a fundamental difference between pattern-matching models and the current one. The atoms (templates) used in this study contain both spatial and temporal activities (Fig 3). Consequently, although the patterns of the AN population activities may be spectrally unresolved, there is still enough spatiotemporal information for identifying the different harmonics (Fig 9). Additionally, the SC does not rely on synthetic bases such as the sine and cosine of the Fourier transform (FT) but actual AN activities. Of course, these bases are also unresolved for high tones which means that the model exhibits less salience with unresolved stimuli, again, in line with psychoacoustic experiments [13]. Note that since the SC model implicitly inherits this property for high tones from the known attributes of the cochlear model, there is no need to add this feature into the model explicitly.

Historically, models of pitches were linked to harmonic analysis theories. Thus, it is instructive to note the mathematical connection between the SC model and the FT. Indeed, for the particular case of deprecating the dictionary matrix **D** into a square matrix, and setting λ = 0, the optimal solution for any periodic signal **x** of Eq 2 is given by the FT. In this case, the optimal solution is for **h** to be the coefficients of the FT and **D** its matrix [62]. In the SC case, applying λ ≠ 0 enables the use of biologically oriented, non-orthogonal, and redundant dictionaries [63]. In this sense, the current proposed model can be seen as an extension of classic pattern-matching models.

How does the SC model compare with temporal ones? Traditional temporal models, the most prominent of which are based on the summary autocorrelation function (SACF), also exploit temporal features from the AN population responses [2,3,10,64]. However, there are several open issues with these type of models. First, SACF models need to have long tapped delay lines for the correlation module, i.e., about 40ms and maybe more if noise is accounted for [15,65]. Currently, however, there is no physiological evidence to support such structures [9,31]. In contrast, the SC model exploits local spatiotemporal features without the need for long tapped delay lines. For that reason and to keep the model biologically plausible, we chose to use only short time segments of 5ms (see [66]). Different temporal interval durations were also tested. For shorter time intervals, e.g., 2ms, the SC model acts as a place-rate model; i.e., it managed to estimate only low resolved signals by their spatial activities along the BM. Longer time intervals also improved the model predictions for unresolved stimuli, up to a maximal estimation of about 10ms. Another issue associated with temporal models is that they treat resolved and unresolved frequencies in a similar manner in contrast to known psychoacoustic measurements [11,12,14,46]. This may imply that tonotopic organization is not necessary for auditory processing, but, again, physiological evidence suggests otherwise [13]. Tonotopic organization is preserved in the auditory system up to the auditory cortex for all mammals [67–70], and current evidence suggests that pitch processing is also sensitive to it [9,14].

Recently, Laudanski et al. [71] proposed a structural theory of pitch that considers both the spatial and the temporal aspects of the AN population response. Within this framework, the perception of pitch is derived from correlated activity in pairs of points in the spatiotemporal representation of AN activities. These two points are not necessarily located along the temporal activity of the same AN fiber (pure temporal processing), nor between different AN fibers at a particular time (pure spatial processing).

Both the structural theory and the proposed SC model are strongly related. In the SC model we incorporate the so-called cross-channel delays of the structural theory in the spatiotemporal patterns of the atoms. Specifically, cross-channel delays of a stimulus are compared to other cross-channel delays of the model that are embedded in the atoms. We think, however, that approaching the problem of pitch estimation from the SC aspect offers considerable benefits. First, the SC approach provides a mathematical framework that generalizes to other modalities whereas the structural theory approach offers a more specific pointwise approach [23]. Second, cross-channel delays of the structural theory can be simply acquired under the SC method by using predesigned atoms (as shown in this paper) or unsupervised training of atoms (see [36,72–74] to name just a few). These techniques were already tested, including within the auditory system [57], with great success. Thus, the SC framework can explain different possible options for such cross-channel correlations.

Other theories and models that exploit the spatial, the temporal, or both, include: the spatial cross-correlation theory of Loeb et al. [75]; de Cheveigne’s solution for the problem of tapped delay lines in temporal theories [65]; Carney’s model of phase-opponency [76], Shamma et al.’s lateral-inhibition and cross-correlation-matrix model [77–79], and the MASD of Cedolin et al. [80]. Note, however, that, these models consider only a small subset of the whole two-dimensional spatiotemporal structure created by the AN fibers. For example, Loeb et al. proposed comparing two locations along the BM that vibrate with the same phase, that is, a spatial comparison without the time domain. De Cheveigne proposed to compensate phase shifts between adjacent cochlear filters, i.e., extracting temporal lags and discarding the spatial information; and Cedolin et al. proposed a model that is based on spatial derivation between cochlear filters with a temporal summation, namely, accounting for the differences between two adjacent cochlear filters and averaging over the time domain. Additionally, these models account well for resolved stimuli but not for unresolved ones [71].

### The Pitch Estimation Module

The use of the harmonic sieve can be considered from different perspectives. First, from the probabilistic point of view it can be seen as an implementation of a likelihood function: the probability of a particular pitch given a set of (parsimonious) coefficients **h**. This approach originates from the pattern-matching theory, and since the proposed model can be seen as an extension to the pattern-matching models, the same theoretical and experimental motivations also apply here. For example, following Goldstein et al. [16], we chose the templates of the harmonic sieve to be Gaussian functions [16,40,58,81]. It might be that for different dictionaries, e.g., dictionaries that contain harmonic stacks, there are better options, but we were not concern with optimizing this feature in this paper.

Second, from the physiological perspective, the harmonic sieve can be thought of as a simple feedforward neural network. In such instance, a set of Gaussian templates of one tone (one row in the matrix G) can be seen as a neuron with a modulated selectivity curve, i.e., a neuron that responds to a particular tone and its successive octaves. For examples of such implementations, see [61,82]. Finally, the harmonic sieve can be considered as a simple (i.e., linear) readout function that extracts the perceived pitch from the activities of the spatiotemporal receptive fields and introduces it in a manner that enables an easy comparison with psychoacoustic data.

Third, from the biological perspective, it had been shown [83] that harmonic templates of this sort can emerge naturally from basic processing in the auditory periphery. Specifically, Shamma et al. demonstrated that the fundamental features include: frequency analysis, fast changing delays at the CFs, phase-locking, and half-wave rectification. All of these properties part of the cochlear models that we used.

### The Dictionary

In this paper, we checked two types of dictionary families: the first is created by sine stimuli while the second is created by stimuli of harmonic tones (i.e., harmonic stacks). These dictionary types were constructed and tested for various amplitude levels. Choosing a dictionary can influence the congruity of the SC model’s results with psychoacoustic measurements: the sensitivity of the model to resolved and unresolved stimuli, the response to low and high stimuli levels, etc. All these features emerge from the cochlear properties that are encapsulated within the atoms. Thus, an important question is which dictionary can be acquired in a biologically compelling manner and will best match psychoacoustic measurements? We intend to investigate this interesting question further in a future paper. However, in this paper we focused on the main premise—that parsimonious representation of auditory information can explain relatively high cognitive tasks, such as that of the percept of a pitch.

Accordingly, we chose to work, for the most part, with a simple dictionary of pure tones. Indeed, pure sinusoidal stimuli are rare in our natural acoustic surroundings [84] and there is no guarantee that the auditory system has access to such components at all. However, note that there is a subtle difference between plausible stimuli for a (hypothetical) training process and the outcome of this process, the atoms themselves. This is an important distinction because it implies that it is reasonable to assume that a learning process over natural-like stimuli, for example, vowels and consonants, can yield local spatiotemporal atoms and not necessarily stack-like atoms. Moreover, there is circumstantial physiological evidence to support similar spatiotemporal structures along different areas in the auditory system. For example, Norman-Haignere et al. [85] researched specific regions in the anterior auditory cortex that strongly react to resolved harmonic tones, and, to a lesser extent, to unresolved ones. Additionally, Carney et al. found, in the anteroventral cochlear nucleus [66], cells that have distinct spatiotemporal tuning patterns in response to pure tones.

Acquiring a dictionary directly from the AN population response is not the necessarily the only implementation nor is it the optimal one. A different approach that has been successfully applied and has many variants [24,34–37] is to perform an unsupervised training from a randomly selected stimulus set that represents sampled statistics of the input domain. These unsupervised learning algorithms create dictionaries that are oriented to specific kind of inputs, for example, natural sounds [73]. Subsequently, they usually generate dictionaries that lead to sparser and more accurate results. It is thus important to realize that the proposed SC technique is not limited to a particular set of atoms (see for example Ch. 12 in [25]).

### Plausible Implementations by Artificial Neural Networks

Throughout this paper, we tried to keep the discussion at the representational level[59]. Accordingly, we did not introduce a plausible neural network mechanism to concentrate on what we saw as the central theme of the current paper—the generalized principle of using sparse representation also for high perception tasks—such as the estimation of pitches. We felt sufficiently confident to follow such a path because the current literature already includes several plausible neural network implementations of sparse coding [86–90] Another important point is that we did not introduce a state-of-the-art solution but a qualitative one. For this reason, the current model operates on stimuli with fixed time intervals. One relatively simple and standard technique to alleviate this restriction is by running the same model in consecutive times (for example, [73]).

### About the Normalization of the AN Response

In the current paper, we normalized all AN population activities. By normalization we mean that we divided each AN simulated result by its maximum response. The normalization that we propose stems from the assumption that relevant information about the pitch is related to the overall spatiotemporal structures of the AN population responses and not their absolute instantaneous rate level. The problem of keeping the estimation of the pitch invariant to the stimulus’ level is due to other deformations and nonlinearities in the AN responses: saturation in the activity of the AN fibers; change in the locations of peaks of ANs population activities; or the relative phases between the different AN fibers [31,45]. It is important to stress that this normalization neither changes nor corrects these effects.

It is worthwhile noting that normalization has been observed across the central neural system in general and in the auditory system in particular [91,92]. This stems from the fact the different modalities need to process a large dynamic range of stimuli, whether it is brightness in the visual sensors or a change of few orders of magnitude in the level of the sound stimulus.

## Conclusion

We showed that sparse coding principles that were successfully applied to other modalities can explain pitch perception. This general approach of a parsimonious representation of the sensory information is the main premise of this paper and this finding resonates with ideas of a canonical computation by the nervous system [23,74,93–98]. Specifically, sparse representation of information can explain neural activity in the visual cortex [24,34,95,99]; the olfactory system of insects [100,101]; and findings in the mammalian auditory cortex [102–105] (however, see [106]). Hopefully, this paper is one small step in searching for such a generalized theory.

## Methods

### The Cochlear Model

We used two types of cochlear models: (i) Slaney's MATLAB auditory toolbox [26] and (ii) the more moderate model of Zilany et al. [27–29]. We used Slaney’s model for most of the simulations in this paper because it is relatively accurate and computationally fast for moderate sound levels. This model, however, does not account for the high amplitude level nonlinearities of the cochlea; for this phenomenon we used Zilany’s et al. model (Figs 5 and 6). In Zilany’s model, the parameters were set as: (1) the outer and inner hair cells were taken to be in healthy condition; (2) the fractional Gaussian noise that is related to the spike rate generated by each AN was approximated in order to save computation time; (3) the model was originally built to match the AN population response of the cat, but we used the built-in option to tune it to the human cochlea [60]; and (4), only high spontaneous rate AN fibers were used.

For both cochlear models, the input stimulus and the simulation of the AN fibers were performed with a sampling rate of F_S_ = 100k Hz; both had the same number of AN fibers (N = 200). The simulations of the AN population responses were performed as follows: a 15ms stimulus, $s_{in}(t)\in R^{T_{a}}$, is constructed and the AN population response is calculated using the chosen model. Finally, the number of samples is T_a_ = 15ms∙F_S_ (rounded to an integer if necessary).

The output of the cochlear model is a matrix of time-samples over the number of AN fibers, i.e., T_a_ × N. This matrix is then truncated to contain only the last 5ms samples, i.e., **S**_*AN*_(*t*,*f*_*CF*_) ∈ *R*^*T*×*N*^ and T = 5ms∙F_S_. Lastly, **S**_*AN*_(*t*,*f*_*CF*_) is normalized by its maximum value (0 < |**S**_*AN*_(*t*,*f*_*CF*_)|<1). Since the model of Zilany et al. has upper and lower bounds on possible simulated CFs, between 125Hz to 20k Hz, all simulations were performed within this frequency interval. Finally, following the usual SPL format, the stimulus levels are introduced into the cochlea in pascal units normalized by the threshold of hearing, i.e., ${\text{g}}_{\text{Pa}}=20\mu \cdot 10^{{\text{g}}_{\text{dB}}/20}$.

### The Sparse Coding Model

Each of the atoms **d**_*j*_ ∈ *R*^*T*⋅*N*×1^, j ∈ [1, M], in **D** are taken as the AN population response to a pure tone. Each **d**_*j*_ is the vector form of the respective AN population response matrix, i.e., the vectorized form. The vectorization is performed using MATLAB’s convention of stacking the matrix’s columns one after the other.

The dictionary matrix **D** = [**d**_1_,…,**d**_*M*_] ∈ *R*^*T*⋅*N*×*gM*^ contains M = 1000 atoms for each group g. The groups are collection of atoms with the same CFs but with different phases (Eq 3). Note that **D** is a rectangular (N∙T ≫ M), highly redundant, matrix. The number of atoms was set arbitrarily by trial-and-error. From our experience, fewer atoms (e.g., M = 250), also yielded reasonable results.

In this paper we chose to implement the SC (Eq 2) by means of the least absolute shrinkage and selection (LASSO) algorithm [32]. We did so because it has a simple implementation, has a relatively acceptable running time, and usually yields good results.

The implementation of the LASSO involves an iterative solution derived by gradient descent. Specifically, the vector **h** is the solution of the following iterated equation:

$$h_{k+1}=\text{soft}\left(h_{k}+\frac{1}{\alpha }D^{\text{T}}\left(v_{\text{AN}}-Dh_{k}\right),\frac{\lambda }{2\alpha }\right).$$ (11)

In this equation, **v**_AN_ is the vectorized form of the AN population response **S**_*AN*_(*t*,*f*_*CF*_) (after vectorization and normalization), and the operator soft(*x*,*T*) = *sign*(*x*)max{0,|*x*| − *T*} is defined for each entry in the vector **x**. The algorithm runs until a convergent criterion is met or until a pre-set number of iterations is exceeded. For the algorithm to converge, the parameter α should maintain a certain condition (*α* ≥ max eig(**D**^T^**D**)). For the simulations in this paper we used the LASSO implementation within MATLAB Inc. [107]. We also created a tweaked version of this algorithm, but there was no substantial difference between the two implementations.

### The Pitch Estimation Unit

The pitch estimation unit is a variant of the known harmonic sieve [58] implementation. It denotes the likelihood of a particular pitch given the sparse coefficient vector **h** ∈ *R*^*M*^. We implemented it as a multiplication between $\tilde{h}\in R^{P}$ and the matrix **G** ∈ *R*^*P*×*P*^ (Eq 4) in which P = 15M, interpolating from the M values of **h**.

Each row in **G** corresponds to a candidate pitch f_*p*_ ∈ [125*Hz*,20*kHz*] and is composed of a set of Gaussian weights at successive harmonics for this particular pitch, i.e.,

$$G_{i,j}=\sum_{k}^{}\exp\left(-{\frac{\left(f_{j}-n_{k}f_{i}\right)}{2\sigma_{i}^{2}}}^{2}\right).$$ (12)

The index *i* is taken from 1 to ⌊(20*k*−125)/*σ*_*p*_⌋. Thus, if the sparse vector **h** has a coefficient that relates to the harmonics of the *f*_*i*_ pitch, multiplying by the matrix **G** would emphasize (give high score) to that entry in the *pdf*. Otherwise, if **h** does not contain harmonics that relate to the *f*_*i*_ pitch, the result would be a low score in the *pdf*. The standard deviations of each of the Gaussian curves in **G** is a function of *f*_*i*_,

$$\sigma_{i}^{}=0.2\cdot ERB\left(f_{i}\right)=0.2\cdot 24.7\left(4.37\;f_{i}+1\right).$$ (13)

In this equation, *ERB* stands for the equivalent rectangular bandwidth of Glasberg and Moore [60]. We tried different variations of *σ*_*i*_, including piecewise curves, all with relatively similar qualitative results. The main constraint was to avoid overlap between the Gaussian distributions and to keep adjacent Gaussian curves wide enough to account for noise in the pitch cues in $\tilde{h}$. Finally, using this scheme we only had to calculate the matrix **G** once, making the algorithm relatively efficient and fast.

### Effect of Different Dictionaries

The dictionary *D*_sine_ ∈ *R*^*T*⋅*N*×*M*^ is constructed with stimuli of one tone. It has N = 300 CF channels and M = 1500 atoms, each formed by a tone stimuli of uniformly selected amplitudes over the interval of 30dB to 70dB SPL (*g*_sine_∼unif(30_*dB*_,70_*dB*_)),

$$s_{\mathrm{sine}}\left(t\right)=g_{\mathrm{sine}}^{}\cdot \sin\left(2\pi \cdot {\text{f}}_{0}\text{k}\cdot \text{t}\right)$$ (14)

The dictionary *D*_*stack*_ has 200 CF channels and 1000 atoms. Each of these atoms contains a stimulus of six consecutive harmonics (1^st^-6^th^); these harmonics have linearly decreasing amplitudes (from 1 to 1/6),

$$s_{stack}\left(t\right)=\sum_{k=1}^{6}\frac{g_{stack}}{k}\cdot \sin\left(2\pi \cdot {\text{f}}_{0}\text{k}\cdot t\right).$$

All of the *D*_*stack*_ atoms are formed by *g*_*stack*_ = 40_*dB*_ *SPL* level stimuli. All simulations were made using Carney's model (Zilany's et al.[27–29]) to account for these high amplitude levels. In order to avoid aliasing in the spatiotemporal domain, the maximum frequency in *D*_*stack*_ is the maximum frequency (20k Hz) divided by the number of harmonics (6).

### Transposed Tones

The transposed tone stimuli in this paper are modulated rectified tones with three carrier waves: f_c,1_ = 4k Hz, f_c,2_ = 6.35k Hz, and f_c,3_ = 10.08k Hz (Eq 9). The modulated tone is given by

$${\bar{s}}_{k}\left(t\right)={\left[\sin\left({2\pi \text{f}}_{0}\cdot t\right)\right]}^{+}*h_{LP,k}\left(t\right).$$ (15)

In this equation, the rectification operator is given as [*x*]^+^ = max(0,*x*). The operation * is a convolution, and *h*_*LP*,*k*_(*t*) is a four-order Butterworth low-pass filter (see Oxenham et al. paper [13] for more details). The cutoff frequencies of the low-pass filter is taken as 0.2f_c,k_ for each of the three modulated frequencies (k = {1,2,3}).

### Iterated Rippled Noise

Following Yost et al. [42], all the stimuli have amplitude levels of 70dB SPL. To account for this amplitude level, all simulations were performed using Carney's model (Zilany et al. [27–29]). To account for the random phases of the stimuli we used *g* = 10 groups (Eq 3). The same simulations with a dictionary that contains no groups, *g* = 1 and *ϕ*_*g*_ = 0, have been slightly noisier but qualitatively the same. Each of the six cases shown in Fig 13 is a histogram of 500 simulations. All the repetition cases are normalized respectively. The maximum peaks of the pdfs are selected within an interval of one octave around the pitch frequency, 1/*d*. All simulations are performed using the same dictionary; this dictionary contains atoms that are 5ms long and have one tone at a level of 45dB SPL. Finally, all stimuli are filtered by a low-pass with a frequency band of 4k Hz [42].

### Musical Notes

We used a dictionary with 1000 sine-atoms of length 5ms. Each such sine-atom composed a group of 10 different phases (g = 10, Eq 3). For these simulations, we used Slaney's MATLAB toolbox [108] as the cochlear model (much faster); the cochlea had 300 CF channels. We used Eq 2 for each of the *T*_*steps*_ = 100 timesteps separately. Each of the SC coefficient vectors $\tilde{h}\in R^{gM}$ was averaged and normalized appropriately into **h** ∈ *R*^*M*^ (each group separately). The collection of all these vectors, for all *T*_*steps*_, formed the matrix $H_{g}\in R^{M\times T_{steps}}$.

Each of these SC vectors (the columns of *H*_*g*_) are then processed by the harmonic sieve to produce the probability of pitch $P_{g}\in R^{M\times T_{steps}}$ at each time step (Fig 14C). Finally, to have one single probability for each stimulus, the matrix *P*_*g*_ is averaged over the time domain and normalized appropriately (Fig 14D). As in previous cases, the pitch of the signal is defined as the maximum point in this pdf.

All measurements were downloaded from the University of Iowa, electronic music studio, from the musical instrument samples page [55].
